# Highly selective cannibalism in the Late Pleistocene of Northern Europe reveals Neandertals were targeted prey

**DOI:** 10.1038/s41598-025-24460-3

**Published:** 2025-11-19

**Authors:** Quentin Cosnefroy, Isabelle Crevecoeur, Patrick Semal, Mateja Hajdinjak, Alba Bossoms Mesa, Johannes Krause, Guido Alberto Gnecchi-Ruscone, Cosimo Posth, Hervé Bocherens, Thibaut Devièse, Hélène Rougier

**Affiliations:** 1 UMR 5199 PACEA, CNRS, Université de Bordeaux, Ministère de La Culture, Pessac, France; 2 Service of Scientific Heritage, Royal Belgian Institute of Natural Sciences, Brussels, Belgium; 3 Department of Evolutionary Genetics, Max Planck Institute for Evolutionary Anthropology, Leipzig, Germany; 4 Department of Archaeogenetics, Max Planck Institute for Evolutionary Anthropology, Leipzig, Germany; 5 Archaeo- and Palaeogenetics, Department of Geosciences, Institute for Archaeological Sciences, University of Tübingen, Tübingen, Germany; 6 Senckenberg Centre for Human Evolution and Palaeoenvironment, University of Tübingen, Tübingen, Germany; 7 Biogeology, Department of Geosciences, University of Tübingen, Tübingen, Germany; 8 CEREGE, Aix-Marseille Univ, CNRS, IRD, INRAE, Collège de France, Aix-en-Provence, France; 9 Department of Anthropology, California State University Northridge, Northridge, CA USA

**Keywords:** Neandertals, Lower limb, Phenotypic diversity, Mobility, Behaviour, Exocannibalism, Ecology, Ecology, Evolution, Zoology

## Abstract

The Troisième caverne of Goyet has yielded the largest assemblage of Neandertal remains in Northern Europe with clear evidence of anthropogenic modifications. However, its skeletal fragmentation has long limited detailed morphological and behavioural study on the assemblage. In this study, we integrate palaeogenetic, isotopic, morphometric, and structural analyses of the long bones to assess the biological profiles of the Neandertals from Goyet and explore whether they present particularities that could shed light on the formation of this unique cannibalised assemblage. We identify a minimum of six individuals, including four adult or adolescent females. Compared to *Homo sapiens* and Neandertals—including regional specimens—the females from Goyet display short statures and reduced diaphyseal robusticity of their long bones. They lack skeletal markers associated with high mobility despite isotopic evidence for non-local origins. The overrepresentation of short, morphologically gracile, non-local females, alongside two immature individuals, suggests a strong selection bias in the individuals present at the site. Dated between 41,000 and 45,000 years ago, a period marked by Neandertal cultural diversity, biological decline and the arrival of *Homo sapiens* in Northern Europe, the cannibalised female and juvenile Neandertals from Goyet indicate exocannibalism, possibly linked to inter-group conflict, territoriality, and/or specific treatment of outsiders.

**Supplementary Information:**

The online version contains supplementary material available at 10.1038/s41598-025-24460-3.

## Introduction

Several occurrences of cannibalistic practices have been identified among Neandertals over an extended time span and across distant geographic regions^1^. The variability in archaeological contexts and assemblages has led to various interpretations of the origins and nature of the behaviours behind cannibalistic practices. Neandertal cannibalism appears to encompass a broad range of motivations, from survival strategies^2^ through commensal acts^3,4^, and to potential ritual contexts^5–7^. However, despite its apparent recurrence and timeframe, interpreting Neandertal cannibalism remains particularly challenging, especially given the fragmentary condition of most skeletal assemblages and the difficulties in assessing the cultural contexts in which these practices occurred.

Within this framework, the Troisième caverne of Goyet offers a unique opportunity to provide new insights into Neandertal behaviours during the Middle to Upper Palaeolithic transition. The early date of the excavation of the Neandertal remains at Goyet (in the 19th and early twentieth century, see Rougier et al*.*^8^) means that field data are missing. However, the site has yielded the largest collection of MIS 3 Northern European Neandertal remains. Taphonomic investigations and microscopic analyses of the remains showed that they exhibit clear signs of anthropogenic modification, including butchery marks (i.e. fresh bone fracturation, percussion notches, and cutmarks) and bone fragments repurposed as retouchers, providing evidence of nutritional cannibalism and suggesting a deliberate human origin for the accumulation of Neandertal remains^8^. The stable isotopic signatures of the Neandertals found at Goyet also indicate that they were non-local, contrasting with the Neandertals from the nearby site of Spy^9^. The presence of several cannibalised individuals with non-local isotopic signatures at Goyet raises questions about the nature, motivations, and dynamics of the interactions among Late Neandertal populations in Northern Europe. However, the highly fragmented nature of the Goyet long bones, which make up most of the assemblage, has so far constrained their morphological analysis and limited comparisons with other fossil human samples.

In this study, we aim to discuss the composition of the Neandertal assemblage of Goyet, as well as evaluate key biological and behavioural aspects. We use a combination of approaches to assess the genetic sex and stable isotopic signatures of the individuals, and to analyse the morphological and (endo)structural properties of their upper and lower limb bones. Our aim is to refine the characterisation of the cannibalised assemblage of Goyet and to explore the potential factors behind its accumulation at the site.

Despite the highly fragmentary nature of the limb bone remains, we were able to complete the biological profiles of the cannibalised individuals through stature estimates and individual robusticity assessment from the long bone length, morphology, and structure. We assessed individual robusticity by using biomechanical indices derived from the cross-sectional geometry (CSG) of the limb bone diaphyses^10,11^. CSG properties were either calculated^12^ or obtained from the literature (Table 1) at different locations on the diaphyses of the radius and lower limb bones. We also explored lower limb structural properties that could be linked with mobility patterns and/or other behavioural specificities. This was done primarily through a landmark-based geometric morphometrics (GMM) analysis of the diaphyseal cross-sectional shape, one of the most informative proxies for inferring behaviour, particularly locomotor and mobility patterns when applied to the lower limb^13^.

**Table 1:** Comparative samples used for the structural properties analyses of the Goyet long bone diaphyses.

Bone	Group	N specimens	Data sources
Radius	Neandertals	6	This study
	UPHS	5	This study
	Neolithic	9	This study
Femur	Neandertals	24	Puymerail et al*.*^26^; Trinkaus and Ruff^27^; Cosnefroy^28^; this study
	MPHS	11	Trinkaus and Ruff^27^; Cosnefroy^28^
	UPHS	75	Trinkaus and Ruff^27^; Ruff^29^; Ruff et al*.*^30^; Cosnefroy^28^; Wei et al*.*^31^; this study
	Neolithic	13	This study
Tibia	Neandertals	17	Trinkaus and Ruff^27^; this study
	MPHS	5	Trinkaus and Ruff^27^
	UPHS	45	Trinkaus and Ruff^27^; Cosnefroy^28^; this study
	Neolithic	9	This study

The cross-sectional shape analysis includes only the specimens from the present study. See Supplementary Table S8 for the detailed composition of the samples. UPHS = Upper Palaeolithic *Homo sapiens*; MPHS = Middle Palaeolithic *Homo sapiens*.

Estimated stature, CSG, and cross-sectional shape analysis of the Neandertals from Goyet were compared to Middle to Late Pleistocene *Homo* specimens, including Neandertals as well as Middle (MPHS) and Upper Palaeolithic *Homo sapiens* (UPHS) (Table 1; Supplementary Table S8). We paid particular attention to comparisons between the Goyet assemblage and relevant samples in terms of chronology and particularly geography, since local topography is known to influence long bone structural properties^13,14^. For this reason, MIS 3 Neandertal specimens from the Meuse Valley sites of Spy and Fonds-de-Forêt were included, as they represent the only material available to assess variability within both a regional and chronological framework^15–18^. In addition, Neolithic specimens from the Meuse Valley were also considered in order to provide a long-term regional perspective^19,20^. Although temporally distant and belonging to a different species, these Neolithic populations retain a strong hunter-gatherer ancestry^21^, making them a relevant comparative group for exploring robusticity and mobility trends in the same terrain. By comparing the specimens found at Goyet to both regional and sub-contemporaneous specimens, we sought to determine the uniqueness of this assemblage among Northern European Late Neandertals and gain insights into the selection criteria and formation processes of the accumulation.

## Results

### Reassessment of the Goyet Neandertal assemblage

The Goyet Neandertal assemblage is composed of 101 skeletal remains (Supplementary Table S1). Two specimens have been added to the inventory through palaeogenetic analysis^22^ since the initial list published in 2016^8^: D183-4, a fragmentary juvenile clavicle, and Q305-1, a neonate femoral diaphysis. Several of the Neandertal remains found at Goyet have been directly dated, including the two new specimens. Here we have calibrated their ages using the presently recommended calibration curve IntCal20^23^ (Supplementary Table S2) since it was published after the initial study of the Goyet Neandertal assemblage^8^. As highlighted in Rougier et al*.*^8^, the age of tooth 2878-2D appears underestimated, and it is also the case for D183-4 and Q305-1^22^, allowing us to refine an age between ca. 41 and 45 ky calBP for the Goyet assemblage (Supplementary Table S2).

As shown by Rougier et al*.*^8^, nearly one-third of the Neandertal specimens from Goyet bear anthropogenic modifications (see also Supplementary Table S1). Cutmarks in particular anatomical areas attest to practices of defleshing and disarticulation, while fresh-bone fractures and percussion notches indicate marrow extraction episodes and attempts. The human assemblage is further characterised by a high frequency of lower limb elements of adult/adolescent individuals, particularly tibiae and femora, which represent the most nutritionally-rich parts of the skeleton^8^. Clavicle D183-4, which belongs to an immature individual, also bears cutmarks, thus extending the evidence of anthropogenic modifications beyond the adult/adolescent limb remains. The processing of the human remains, along with their use as retouchers and the selective anatomical representation are comparable to those of the associated fauna, mainly horse and reindeer, and correspond to the criteria of nutritional cannibalism^24,25^, similar to what has been documented at the Middle Palaeolithic site of Moula-Guercy^3^.

Previous analyses had revealed the sex of one of the Neandertal femurs (Femur I) to be female through nuclear genome sequencing of one of its fragments (Q56-1)^32^. In the present study, the genetic sex of seven additional specimens was determined to be female (Q55-4—Tibia IV, Q57-1—Tibia II, Q57-2—Femur II, Q57-3—Tibia III, Q305-4—Tibia I, tibia specimen Q305-7, and Q374a-1—Tibia V) and that of the juvenile clavicle D183-4 and of the neonate femur Q305-1 to be male (see Methods and Supplementary Data 1). In addition, we analysed the stable isotopic ratios *δ*^13^C and *δ*^15^N of the collagen from the two newly identified Neandertal specimens (D183-4 and Q305-1) in order to complement the data previously obtained from other Neandertal remains at Goyet^9,33^ (see Supplementary Data 2). The new ratios fall within the variation of the other Neandertal specimens from Goyet (Supplementary Fig. S2).

The initial morphometric, taphonomic, and palaeogenetic analyses of the assemblage indicated the presence of a minimum of five Neandertals, four adolescents/adults and one child represented by a single tooth (1424-3D)^8^, while the present study has revealed a minimum of six individuals. Further palaeogenetic analyses, and in particular the retrieval of nuclear DNA from seven specimens, revealed an erroneous fragment refitting and allowed to associate to the same individuals several femur and tibia specimens lacking direct anatomical connections^34,35^. This leads to identify three Goyet Neandertal (GN) adult/adolescent females: GN1, composed of right Femur I (thereafter GN1-FemI), tibia fragment Q305-7, and right Tibia V (GN1-TibV); GN2, with right Femur II (GN2-FemII), right Tibia III (GN2-TibIII), and left Tibia II; and GN3, made of left Tibia I (GN3-TibI). A fourth individual (GN4) corresponds to the child represented by tooth 1424-3D. The newly identified clavicle D183-4 has a stage of development that is compatible with belonging to the same ~ 6.5–12.5 year-old child^8^, and in order to propose a true minimum number of individuals, we will refer to it as belonging to individual “GN4?”. Individual GN5 is a male neonate represented by the femoral diaphysis Q305-1. Finally, one additional adult/adolescent is identified: the female GN6, represented by right Tibia IV (GN6-TibIV), which preserves overlapping areas with the right tibias of GN1 and GN2, and whose *δ*^34^S value^9^ is too different from that of GN3-TibI to belong to the same individual^36^.

In order to assess the singular demographic composition of the Goyet assemblage while taking into account the small size of the sample, we used a resampling procedure to test the probability of randomly drawing the individual representation found at Goyet (six individuals, four adult/adolescent females and two juveniles) from theoretical mortality profile models^37^, as well as from a population reflecting the individual representation pattern of the Neandertal assemblage from Chagyrskaya^38,39^ (Supplementary Data 6, Supplementary Table S10). Across all simulations, the probability of replicating the composition observed at Goyet is either statistically highly unlikely (*p* < 0.01 under the Q25 model and the Chagyrskaya model with the largest MNI) or statistically unlikely (*p* < 0.05 under the Q30-Q35 models and the Chagyrskaya model with the lowest MNI) (Table 2). In contrast, the El Sidrón cannibalised assemblage is much more likely to occur under random draws, particularly when compared to the individual representation pattern of the Chagyrskaya assemblage, which in turn aligns well with the theoretical mortality profile model of a population with a life expectancy at birth of 35 years. These results suggest that the composition of the Goyet assemblage reflects a deliberate selection of individuals rather than random, size-affected sampling of a few individuals from a population following a natural mortality profile.

**Table 2:** Frequencies from 10,000 random draws (with (R) and without (noR) replacement) with which the observed composition of the Goyet, El Sidrón, and Chagyrskaya Neandertal individual assemblages occur in reference populations of 60 individuals following theoretical mortality profiles reported by Ledermann^37^ and the profile documented in the Chagyrskaya Neandertal assemblage^38,39^.

Target assemblage	Assemblage composition (F/M/JUV)	Draws	Ledermann Q25	Ledermann Q30	Ledermann Q35	Chagyrskaya (N = 12)	Chagyrskaya (N = 11)
Goyet	N = 6 (4/0/2)	R / noR	0.004** / 0.008**	0.013* / 0.013*	0.016* / 0.018*	0.008** / 0.001**	0.016* / 0.012*
El Sidrón	N = 13 (3/4/6)	R / noR	0.027* / 0.027*	0.064 / 0.053	0.072 / 0.055	0.074 / 0.063	0.069 / 0.062
El Sidrón	N = 13 (4/3/6)	R / noR	0.027* / 0.026*	0.070 / 0.056	0.076 / 0.056	0.051 / 0.047*	0.073 / 0.058
Chagyrskaya	N = 12 (3/4/5)	R / noR	0.020* / 0.020*	0.056 / 0.047*	0.073 / 0.064	–	–
Chagyrskaya	N = 11 (3/3/5)	R / noR	0.041* / 0.040*	0.085 / 0.066	0.091 / 0.071	–	–

F/M/JUV refer to the numbers of adult/adolescent females, adult/adolescent males, and juveniles (0–14 years old). *p < 0.05; **p (in bold) < 0.01. Qxx indicates different life expectancies at birth. See Supplementary Data 6 for details on each sample.

**Fig. 1:**
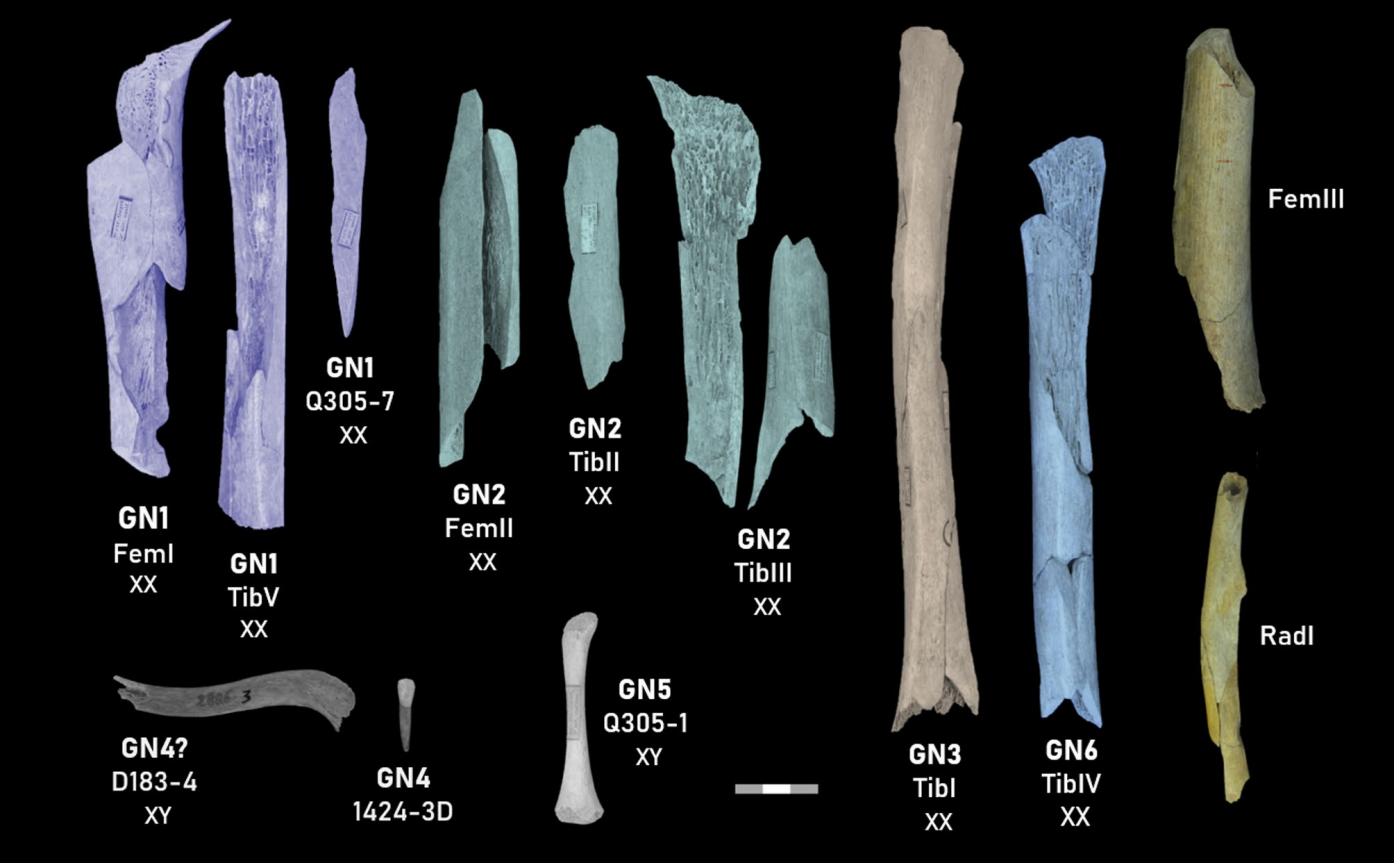
Neandertal specimens from the Troisième caverne of Goyet included in this study. Genetic sex determinations: XX indicates female, XY indicates male. Specimens belonging to the same individual are shaded in the same colour.

The next step of the study consisted in completing the biological profiles of the Neandertals from Goyet by estimating individual stature and diaphyseal robusticity and inferring behavioural patterns from the lower limb structural properties. We reviewed the preservation of the Goyet long bones from the upper limb (humerus and radius) and lower limb (femur and tibia) (Supplementary Data 3) to select the specimens that were sufficiently preserved for the analyses (see Methods). They belong to the upper limb (GN-RadI) and lower limb (GN1-FemI, GN1-TibV, GN2-FemII, GN2-TibIII, GN3-TibI, GN6-TibIV, and GN-FemIII) (Fig. 1). They represent a minimum of four adult/adolescent females while two of the specimens (GN-RadI and GN-FemIII) have not been associated to any of the identified GN individuals and neither have been assigned a sex (Table 3; Supplementary Table S1).

**Table 3:** Descriptive data of the Goyet long bones included in this study and individual stature estimations using Sjøvold^43^ formulae on tibial or femoral MaxL, with the theoretical error ranges provided by Sjøvold’s equations^43^ for the statures estimated in this study (Supplementary Data 4). Specimens included in the calculation of the Neandertal mean and standard deviation: Neandertal 1^a^, Palomas 96^a^, Shanidar 2^a^, Shanidar 4^a^, Shanidar 5^a^, Shanidar 6^a^, Tabun 1^a^, Amud 1^a^, Ehringsdorf E^b^, Fonds-de-Forêt 1^a^, Hohlenstein 4^b^, La Chapelle-aux-Saints 1^a^, La Ferrassie 1^a^, La Ferrassie 2^a^, La Quina 5^b^, Spy 8^a^, CDV-Tour 1^c^. ^a^Femoral MaxL from Trinkaus and Ruff^27^; ^b^Femoral MaxL from Cosnefroy^28^; ^c^Femoral MaxL derived from Puymerail et al.^26^, using MaxL = BML × 1.037 + 11.2; *For a discussion of the sexing of Neandertals in the absence of genetic data, see Supplementary Data 5.

Specimen	Sex	MaxL (mm)	BML (mm)	Estimated stature (cm)
GN-RadI	ND	ND	ND	–
GN1-FemI	F	408	383	156 ± 4.49
GN2-FemII	F	ND	ND	–
GN-FemIII	ND	398	373	154 ± 4.49
GN1-TibV	F	306	282	–
GN2-TibIII	F	324	303	153 ± 4.15
GN3-TibI	F	271	292	143 ± 4.15
GN6-TibIV	F	313	292	150 ± 4.15
Mean Goyet				151.56 ± 5.05
Mean Neandertals (N = 17) local Neandertals Spy 8; Fonds-de-Forêt 1 female Neandertals* Palomas 96; La Ferrassie 2				162.89 ± 8.20 159 ± 4.49; 174 ± 4.49 147 ± 4.49; 157 ± 4.49

### Stature estimation

Individual stature was estimated using the maximum length of the lower limb long bones (Table 3). All Neandertals from Goyet present shorter estimated statures than the Neandertal average (Welch’s t-test: *p* = 0.001; Supplementary Table S9). GN3 even falls below two standard deviations of the Neandertal range of variation, close to the presumed female Palomas 96^40^ (Supplementary Data 5). GN-FemIII of unknown sex also exhibits a moderately short estimated stature that is similar to that of the female GN2 and comparable to the presumed female La Ferrassie 2^41,42^. The other Northern European Neandertals—Spy 8 and especially Fonds-de-Forêt 1—both present larger statures than the Neandertals from Goyet.

### Structural properties

The structural properties of the long bone diaphyses, through CSG and GMM analysis of their cross-sectional shape, reveal notable patterns in individual robusticity, mobility, and biological affinities of the Goyet specimens among Neandertals.

**Fig. 2:**
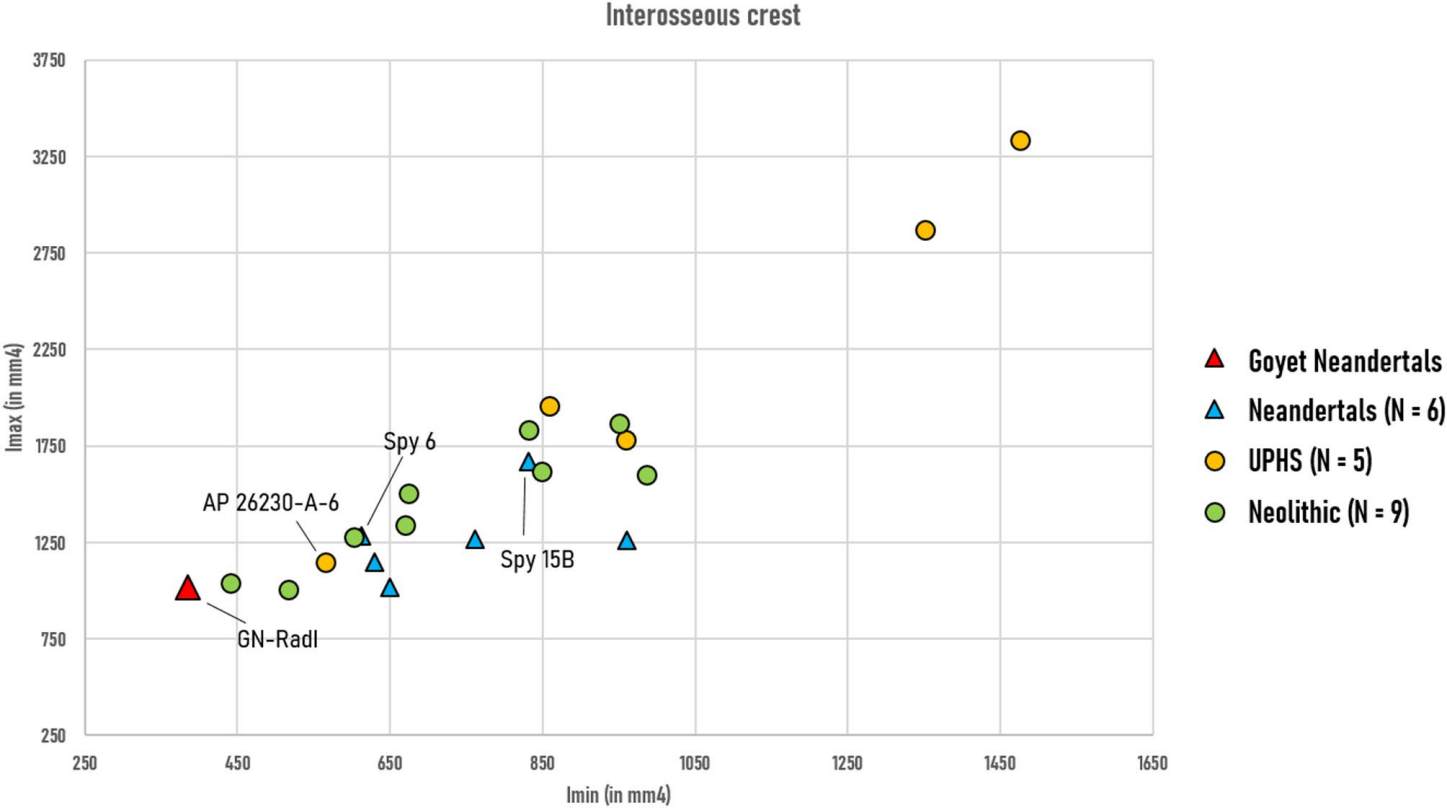
Radial maximum (Imax) vs minimum (Imin) second moments of area at the level of the maximum medial extension of the interosseous crest. This measurement allows to distinguish homogeneous (Imax/Imin ~ 1) vs heterogeneous (Imax/Imin > > 1) cortical distributions. AP 26230-A-6: Abri Pataud 26230-A-6.

GN-RadI stands out compared to other Neandertals by having the lowest values of second moments of area (Imax, Imin), indicating reduced hypertrophy, while its low robusticity index (Zp) indicates diaphyseal gracility at the maximal extension of the interosseous crest (Fig. 2, Table 4). The UPHS specimens except for one (Abri Pataud 26230-A-6) exhibit higher Imax and Imin values than both the Neolithic and Neandertal specimens, indicating increased radial hypertrophy in this group. The GMM cross-sectional shape analysis does not indicate any distinct patterns between the comparative groups, with GN-RadI falling within the Neandertal cross-sectional shape variability (Supplementary Fig. S3).

**Table 4:** Cross-sectional geometry properties of the Goyet long bones compared to the Neandertal variation (mean + /- sd).

Bone	Preserved cross-section location*	Specimen	CA (in mm^2^)	TA (in mm^2^)	Ix (in mm^4^)	Iy (in mm^4^)	Imax (in mm^4^)	Imin (in mm^4^)	Zp (in mm^3^)
Radius	IC**	GN-RadI	76.38	86.63	384.05	1009	1014	385	144
		Neandertals (N = 6)	93.70 ± 7.78	110.27 ± 8.81	750.53 ± 138.31	1,265 ± 224.64	1,274 ± 217	740 ± 137	242 ± 44.87
Femur	80%	GN1-FemI	419.86	62.55	24,109	30,175	34,853	19,435	3004
		Neandertals (N = 17)	541.58 ± 94.37	737.10 ± 124.79	38,229 ± 12,068	45,652 ± 13,761	50,793 ± 13,553	33,086 ± 12,236	4665 ± 991.46
	50%	GN2-FemII	452.13	591.99	22,223	31,205	31,236	22,228	3480
		Neandertals (N = 23)	502.43 ± 90.88	651.37 ± 108.23	32,317 ± 11,219	33,896 ± 9,678	37,784 ± 11,854	28,430 ± 8920	4238 ± 604.90
	35%	GN-FemIII	403.58	609.11	25,715	26,901	29,747	22,925	3,513
		Neandertals (N = 12)	451.45 ± 56.04	703.91 ± 50.65	35,833 ± 7489	33,288 ± 4439	38,029 ± 8554	28,723 ± 4999	4166 ± 507.36
Tibia	50%	GN6-TibIV	276.96	405.92	14,333	10,597	16,568	8380	1721
		Neandertals (N = 15)	383.26 ± 67.33	509.80 ± 82.33	28,716 ± 11,505	14,554 ± 4605	30,379 ± 11,675	13,135 ± 3915	2,894 ± 171.20
	35%	GN3-TibI	260.18	391.35	11,937	10,749	13,961	8749	1620
		GN7-TibIV	260.05	383.49	12,069	9666	13,384	8345	1643
		GN1-TibV	274.69	401.12	13,117	10,581	13,469	10,207	1722
		Neandertals (N = 9)	351.58 ± 77.73	484.16 ± 88.28	21,732 ± 8342	15,651 ± 5818	23,725 ± 9452	13,655 ± 4683	2400 ± 86.99
	20%	GN3-TibI	164.02	461.87	9318	11,169	11,169	9269	1385
		Neandertals (N = 8)	261.49 ± 50.25	516.92 ± 90.61	15,789 ± 5813	17,525 ± 5013	19,167 ± 5978	14,178 ± 4750	2534 ± 226.91

*The percentages refer to the section positions relative to the bone biomechanical length. **IC: maximum medial extension of the radial interosseous crest.

**Fig. 3:**
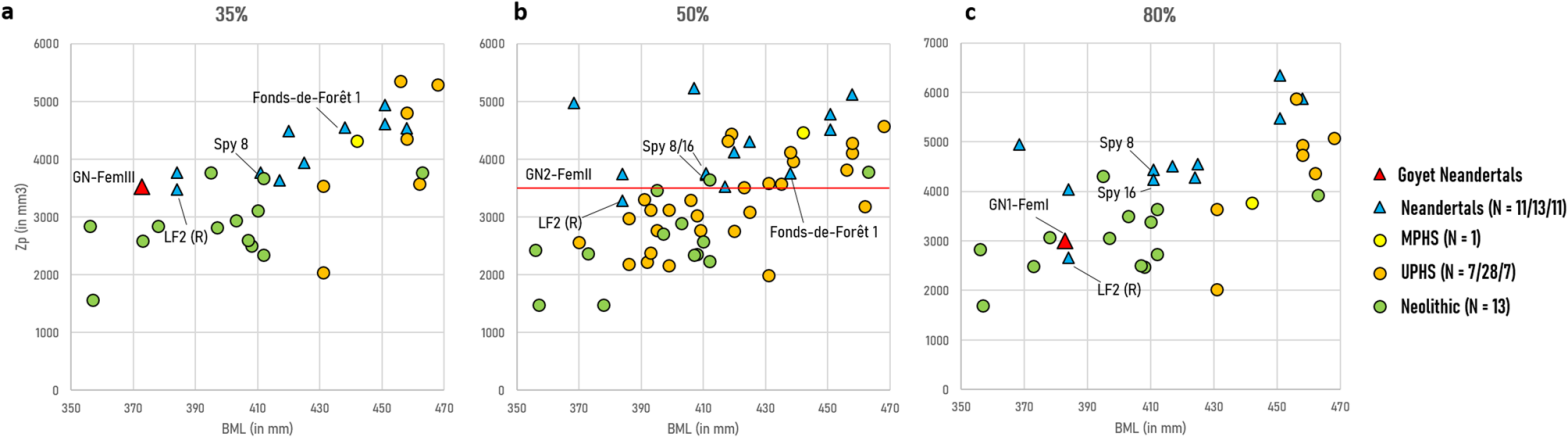
Femoral polar section modulus (Zp) vs biomechanical length (BML) at (**a**) the mid-distal (35%), (**b**) midshaft (50%), and (**c**) subtrochanteric (80%) cross-section locations. The BML of GN2-FemII could not be estimated due to preservation. LF2 (R): La Ferrassie 2 (Right).

The three Neandertal femora from Goyet present low robusticity indices (Zp) in comparison to other Neandertals, even when considering their moderately short biomechanical length (BML) (Fig. 3, Table 4). Only the right femur of La Ferrassie 2 has lower Zp values, while both the Spy and Fonds-de-Forêt 1 femora consistently present higher values than the Goyet specimens at each cross-section location. At the subtrochanteric location (80% of the BML), GN1-FemI exhibits such a low robusticity that it falls among the Neolithic specimens.

**Fig. 4:**
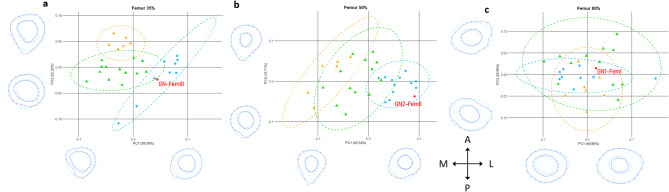
Femoral cross-sectional shape analysis based on Procrustes coordinates from the endosteal and periosteal contours at (**a**) the mid-distal (35%), (**b**) midshaft (50%), and (**c**) subtrochanteric (80%) locations. The wireframes represent landmark configurations for PC scores of 0.1 (right on PC1 and top on PC2) and − 0.1 (left on PC1 and bottom on PC2). Red diamonds: Neandertals from Goyet; blue diamonds: Neandertals (N = 9/12/8); orange triangles: UPHS (N = 6/6/7); green triangles: Neolithic individuals (N = 13/13/12). Ellipses represent the 95% confidence intervals of the comparative groups. LF2: La Ferrassie 2. A: anterior, L: lateral, P: posterior, M: medial.

The GMM cross-sectional shape analysis reveals distinctive patterns between the Goyet specimens and other Northern European Neandertal femora (Fig. 4). For instance, GN1-FemI exhibits a large cortical area in the subtrochanteric region and falls at the opposite end of the Neandertal variation compared to the position of the Spy and Fonds-de-Forêt specimens (along PC1 at 80% of the BML), with only the MIS 5 Ehringsdorf specimen falling next to GN1-FemI. GN2-FemII shows stronger midshaft postero-medial reinforcement than both the Spy and Fonds-de-Forêt 1 femora (PC1 at 50% of the BML). More generally, the mid-distal (35%) and midshaft (50%) femoral cross-sectional shapes are particularly distinctive between all samples (Fig. 4). Neandertals, including the Goyet specimens, exhibit increased mid-distal and midshaft posteromedial reinforcements that distinguish them from the anteroposteriorly-elongated sections of the *Homo sapiens* samples (along PC1 at 35% of the BML and PC1 at 50% of the BML). Among *Homo sapiens*, the UPHS specimens present increased mid-distal posterior reinforcement compared to the Neolithic individuals (along PC2 at 35% of the BML). Therefore, Neolithic femora tend to present intermediate cross-sectional shapes between UPHS and Neandertals.

**Fig. 5:**
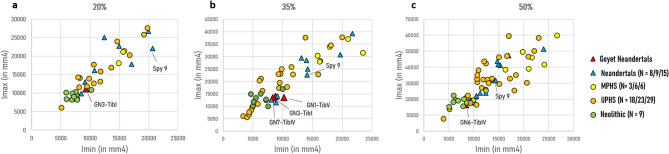
Tibial maximal (Imax) vs minimal (Imin) second moments of area at (**a**) the distal (20%), (**b**) mid-distal (35%), and (**c**) midshaft (50%) cross-section locations. This measurement allows to distinguish homogeneous (Imax/Imin ~ 1) vs heterogeneous (Imax/Imin > > 1) cortical distributions.

The Neandertal tibiae found at Goyet present low Imax and Imin values at each cross-sectional location, revealing reduced tibial hypertrophy and slender lower limb extremities. They also exhibit relatively low Imax/Imin ratios compared to UPHS, MPHS and other Neandertals, suggesting increased cross-sectional circularity and homogeneous cortical distributions (Fig. 5).

**Fig. 6:**
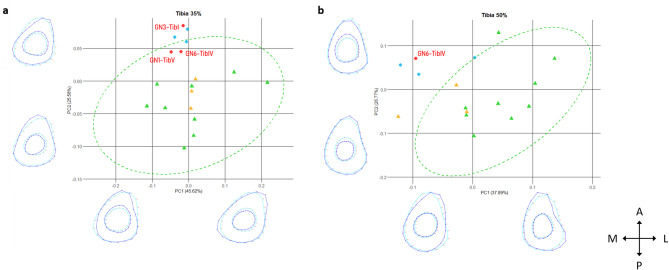
Tibial cross-sectional shape analysis based on Procrustes coordinates from the endosteal and periosteal contours at (**a**) the mid-distal (35%) and (**b**) midshaft (50%) locations. The wireframes represent landmark configurations for PC scores of 0.1 (right on PC1 and top on PC2) and − 0.1 (left on PC1 and bottom on PC2). Red diamonds: Neandertals from Goyet; blue diamonds: Neandertals (N = 3); orange triangles: UPHS (N = 3); green triangles: Neolithic individuals (N = 9). Ellipses represent the 95% confidence intervals of the comparative groups. LF2: La Ferrassie 2. A: anterior, L: lateral, P: posterior, M: medial.

The GMM cross-sectional shape analyses at midshaft (50% of the BML) and particularly at the mid-distal location (35% of the BML) illustrate a particular morphology for Neandertals, which includes the Goyet and Spy tibiae and differs from the cross-sectional shape observed in the *Homo sapiens* samples (Fig. 6). Specifically, the Neandertal pattern is characterised by reduced cortical areas and increased sectional circularity at the mid-distal and midshaft locations compared to most specimens of both the UPHS and Neolithic samples (along PC2 at 35% of the BML and PC1 at 50% of the BML). Within *Homo sapiens*, UPHS and Neolithic samples differ at midshaft, where the UPHS sample exhibits more homogeneous cortical distributions between the lateral and medial sides of the tibiae (along PC1 at 50% of the BML).

## Discussion

In Neandertals, the femoral diaphyseal robusticity does not correlate with environmental factors^44^ as much as it does among *Homo sapiens*^13,45,46^, suggesting that Neandertal bony structure may have been less responsive to mechanical loading and more genetically determined compared to *Homo sapiens*. However significant differences in diaphyseal robusticity are present between fossil remains attributed to males and females^44,47^. Taken together, this implies that diaphyseal robusticity in Neandertals allows for broad comparisons between specimens from different environmental contexts and supports its use in sex estimation of incomplete specimens. In this study, the three Neandertal femora from Goyet assessed for their structural properties exhibit gracile morphologies compared to other Neandertal specimens, except Palomas 96 and La Ferrassie 2. The robusticity indices (Zp) of femora GN1-FemI and GN2-FemII, genetically sexed as females, fall at the lower end of the Neandertal range, which appears to form a continuum from gracile to more robust morphotypes. Interestingly, the GN-FemIII specimen of unknown sex also displays an extremely low robusticity index, and its cross-sectional shape closely resembles that of the presumed female La Ferrassie 2. The combination of a short estimated stature, gracile morphology, and structural similarity with the genetically-sexed female specimens from Goyet suggests that GN-FemIII may also belong to a female individual. Regarding the tibia, two distinct groups can be identified among Neandertals based on second moments of area (Imax and Imin) values, particularly at midshaft. All three analysed Goyet specimens, genetically sexed as females, cluster with La Ferrassie 2 and Palomas 96, while La Ferrassie 1, La Chapelle-aux-Saints 1, and Amud 1, all proposed males by Rmoutilová et al*.*^42^, fall on the opposite end of the variation spectrum. This pattern suggests that tibial hypertrophy may reflect sexual dimorphism in Neandertals, with females exhibiting less pronounced hypertrophy than males. Interestingly, the Spy 9 tibia displays intermediate values and cannot be confidently assigned to either group. Altogether, the results of the study point to all of the adult/adolescent female individuals of the Goyet Neandertal assemblage being gracile and of short stature. This raises the possibility of a strong selection for adult/adolescent individuals based on their sex, stature, and robusticity in the remains accumulated at the site.

The cross-sectional properties of lower limb bones correlate with locomotor and mobility patterns in Pleistocene hominins. For instance, in the femur of *Homo sapiens*, an anteroposteriorly elongated cross-sectional shape associated to anteroposterior cortical reinforcement indicates high mobility^46,48–51^. Decrease in these features through time has been linked to a reduction of mobility associated to the transition from mobile foraging lifestyles to more sedentary ones from the Late Upper Palaeolithic and during the Holocene^49,52,53^. However, the femoral anteroposterior reinforcement at the origin of this distinctive cross-sectional shape is an autapomorphic feature of *Homo sapiens*, hypothesised to be the result of body proportions and particularly a reduction of the pelvic breadth compared to other Pleistocene hominins^27,54^. Therefore, femoral cross-sectional shape has limited value in inferring mobility behaviour at the inter-specific level or beyond the scope of *Homo sapiens*^55^. In contrast to the femur, the tibial diaphysis consistently displays a triangular cross-sectional shape across populations and hominin species, and can be considered as a more direct indicator of locomotor activity^55^. It has therefore been proposed as a more reliable proxy than the femoral diaphysis for assessing mobility patterns at both the intra- and the inter-specific levels in Pleistocene hominins^27,56–59^. However, no comprehensive study has been conducted to date to discuss mobility patterns among Neandertals, likely due to the limited availability of 3D comparative data^60^. In this study, all four analysed Goyet tibiae present cross-sectional shapes similar to the rest of the Neandertal sample, including the local specimen Spy 9, suggesting a restricted variability in Neandertals for this property. All Neandertals present circular cross-sections compared to *Homo sapiens* samples. This morphology is generally interpreted as a skeletal response to multidirectional loading regime^61^. In contrast, a more elongated cross-section, particularly in the anteroposterior plane, is typically associated with unidirectional loading, such as that seen in habitual runners, and is often observed in highly mobile *Homo sapiens* populations^48,50,61^. Additionally, the tibial diaphyseal hypertrophy reflected in the Imax and Imin values suggests that the Goyet specimens, along with most of the Neandertals, experienced a loading regime with less constraints than both the UPHS and MPHS groups, possibly reflecting reduced mobility patterns in Neandertals compared to Pleistocene *Homo sapiens*. Furthermore, although terrain is widely recognised as a significant factor influencing bony structure^13,14^, our results show that the local Spy 9 tibia differs markedly from the Neolithic sample. This suggests that, in this case, the local topography of the Meuse Valley exerted limited influence on tibial morphology when comparing *Homo sapiens* and Neandertals. Instead, the differences in tibial structure highlighted by our results may reflect variation in the intensity and repetitiveness of daily locomotor activities, and all Neandertals, including the specimens from Goyet, exhibit a structural pattern consistent with lower mobility compared to *Homo sapiens* individuals, including Neolithic ones to some extent^53,62,63^.

Beyond these biomechanical considerations, the consistent differences in long bone cross-sectional shape between Neandertals and *Homo sapiens* samples reinforce the taxonomic value of postcranial morphology and structure^64^ although our study did not reveal any evident trends for the radius. Within Neandertals, our results on the femoral cross-sectional shape indicate biological affinities and intra-specific variability. The MIS 5e Ehringsdorf femur exhibits a mediolaterally elongated proximal diaphysis that resembles the plesiomorphic condition observed in Middle Pleistocene hominins^58,65^, a morphology distinct from that of later Neandertals such as those found at Goyet who align with other MIS 3 specimens. Finally, our study highlights the importance of including fragmented remains for interpretating past population behaviour and for acquiring new insights into postcranial phenotypic diversity of Pleistocene hominins.

Despite being identified as non-local^9^, the Neandertals from Goyet do not exhibit the particular skeletal adaptations typically associated with high mobility. Instead, the structural properties of their long bones indicate low to moderate locomotor activities, which does not support the idea of mobility behaviours having led them away from their main foraging area. Moreover, both their structural properties and estimated statures align with those typically observed in female Neandertals. This is in line with the genetic sex identifications of three of the individuals and it strongly suggests that the fourth adult/adolescent was a female as well. Although female exogamy behaviour has been documented among Neandertal groups^39,66^, the significant demographic anomaly exhibited by the Goyet sample therefore requires revisiting the accumulation processes responsible for the Goyet Neandertal assemblage as a whole.

In a previous study, the Neandertal assemblage from Goyet was shown to exhibit clear signs of anthropogenic modifications, in particular on the infra-cranial skeleton^8^ (Supplementary Table S1). In addition, sulphur isotopic composition analyses of the collagen of sixteen of the bones, including all of those analysed in the present study except for GN-RadI and GN-FemIII, were conducted^9^ (Supplementary Table S5). With all of them representing adult/adolescent individuals and most bearing anthropogenic modifications (one of three rib fragments, clavicle fragment Q55-1, one of two humerus fragments, and all nine lower limb specimens), their sulphur isotopic values indicate a non-local, but similar, origin for the cannibalised individuals. The present morphometric analyses of the femoral and tibial remains of the Neandertals from Goyet have also highlighted their phenotypic homogeneity. They belong to four different gracile adult/adolescent individuals that are characterised by short statures and do not show particular signatures of high mobility pattern. Their genetic analyses have revealed that these individuals are female Neandertals who do not have close kinship relationships^34,35^. Two male individuals are also present in the Goyet assemblage, but they represent a child (GN4?) and a neonate (GN5). Although the neonate GN5, represented only by the sub-complete femoral diaphysis Q305-1, does not bear anthropogenic marks, cutmarks have been identified on clavicle D183-4 of child GN4? (Supplementary Table S1). Carbon and nitrogen isotopic data have also revealed a homogeneous diet across all individuals (see Wißing et al*.*^33^ and Supplementary Data 2), suggesting that they likely belonged to a single social group— or to different groups that happened to share similar diets. Furthermore, similar sulphur isotopic signatures between individuals suggest a single geographic provenance even if not identified. In this context, we can hypothesise that the Neandertal individuals from Goyet that were anthropogenically processed not only indicate exocannibalistic practices^9^, but they also testify to a targeted predatory behaviour toward gracile, short-statured female individuals, and possibly immature individuals along them.

In the ethno-archaeological record, exocannibalism is typically associated with warfare or competition between groups, involving the violent abduction of individuals from outside communities^67–72^. In such cases, exocannibalism is consistently documented as part of collective inter-group violence, with butchered remains subsequently repurposed as symbolic objects or trophies^73–75^. In the archaeological record, evidence for predatory behaviours targeting juveniles appears as early as the Lower Pleistocene and has been associated to nutritional cannibalism practices^24,76^. At Gran Dolina, for instance, the targeted selection of immature individuals has been compared to inter-community violence and cannibalism among chimpanzees, which generally occurs in the context of territorial or resource-related conflicts^24^. At Goyet, the unusual demographic mortality profile of the cannibalised individuals (adolescent/adult females and young individuals) cannot be considered natural, nor can it be explained solely by subsistence needs, especially given the abundant associated faunal remains that show similar butchery marks. At a minimum, it suggests that weaker members of one or multiple groups from a single neighbouring region were deliberately targeted. It can also be hypothesised that this selection strategy might have aimed at undermining the reproductive potential of (a) competing group(s) as proposed for Gran Dolina^76^. Comparable behaviours have been documented in multiple contexts of inter-group conflict since the Neolithic^67,74,77,78^. Although the precise causes of inter-group tensions in Pleistocene contexts remain difficult to establish^79^, the regional chronocultural context is consistent with the hypothesis that conflict between groups played a role in the accumulation of the cannibalised individuals at Goyet.

Indeed, even if *Homo sapiens* groups are not (yet) documented in the region at the same time as Neandertals are at Goyet, they are present roughly contemporaneously at Ranis, Germany, about 600 km to the east^80–82^. In addition, cultural variations are well documented in Northern Europe within Middle Palaeolithic lithic traditions. The Belgian territory along the Sambre-Meuse axis appears as a fluctuating corridor between Western European Mousterian-type industries and Central and Eastern European “Keilmesser group” traditions, the latter illustrated by the site of Feldhofer, about 100 km east from Goyet^83,84^. These strong cultural differences might indicate limited interaction between contemporaneous Neandertal groups that could have perceived each other as different. With the gradual arrival of new *Homo sapiens* groups, who appear not to have interacted with local Neandertals^80^, demographic pressure and group competition might have surged in the region.

Therefore, it raises the question of whether the Goyet predatory group was composed of Neandertals or early *Homo sapiens*. It is theoretically possible to suggest that the cannibalised Neandertals at Goyet were selected by early *Homo sapiens* groups associated to the LRJ since the site preserves tenuous evidence of this techno-complex^85^. Furthermore, various forms of cannibalism have been documented at later European Upper Palaeolithic sites^1^. However, in many of these cases, this practice appears to be connected to funerary treatments, with the skeletal assemblages and/or the anthropogenic modifications differing from butchery patterns typically observed on faunal remains^86,87^. In more ambiguous prehistoric cases where clear nutritional cannibalism is present, the assemblages consistently exhibit evidence of ritual traits with the symbolic use of human remains, particularly of cranial elements^74,88–91^. Finally, no human bone retoucher has ever been identified in such Upper Palaeolithic contexts, whereas this practice has been documented across time and space in other cannibalistic contexts where the authors could only have been Neandertals^4,6,92^. As a result, while the *Homo sapiens* predator hypothesis cannot be entirely ruled out, we consider the hypothesis of inter-Neandertal group behaviour to be the more likely explanation for the assemblage accumulation at Goyet.

If the causes leading to cannibalistic behaviours are always difficult to establish in archaeological contexts, the integrated approach developed here—combining taphonomic, isotopic, genomic, and morphometric data— provides an unprecedented characterisation of the Goyet assemblage. Nutritional cannibalism practices, which are archaeologically indistinguishable from the butchery activities observed on the associated fauna, were performed on selected Neandertal individuals: short-statured, gracile female adults/adolescents, and juveniles who were not closely related but shared similar isotopic signatures indicating comparable diets and geographic origins. The demographic pattern, skeletal representation, and butchery marks differ from the similarly well-documented case of endo-cannibalism at El Sidrón^2^. Instead, the case of Goyet represents the most compelling evidence to date for inter-group competition among Late Pleistocene Neandertal populations.

## Methods

### Study of the Goyet Neandertal assemblage

The Goyet Neandertal collection was observed macroscopically and high-resolution microtomographic scans were performed on a majority of the long bone specimens at the Royal Belgian Institute of Natural Sciences (RBINS), producing scan resolutions with an isotropic voxel size ranging from 27 to 51 µm (see Supplementary Table S8 for details). The preservation and stages of development of the upper and lower limb long bones were briefly described (Supplementary Data 3) and used to select the specimens suitable for further study. Only the specimens whose developmental stages had reached at least adolescence were considered, and we selected the lower limb bone specimens that preserve enough anatomical landmarks to estimate their original lengths and determine the standardised placement of cross-sections on them. These landmarks were identified through a combination of direct observations and virtual reconstructions. For the long bone structural analysis, we selected all upper and lower limb specimens that present at least one fully preserved diaphyseal contour (Supplementary Table S1).

The comparative samples were assembled by combining published data and data from existing (micro)CT scan databases or directly provided by collaborators and institutions (see Supplementary Table S8).

### Mortality profile of the Goyet assemblage

To test whether the Goyet assemblage reflects a selection bias, we statistically evaluated the probability of obtaining its observed composition: four adult/adolescent females, no adult/adolescent males, and two juveniles. We used a resampling procedure consisting of 10,000 iterations of random draws from reference populations (N = 60) that reflect theoretical demographic patterns. Two types of references were used: (1) theoretical mortality profiles derived from Ledermann’s mortality tables^37^ and corresponding to populations with life expectancies at birth of 25 (Q25), 30 (Q30), and 35 (Q35) years; and (2) two Middle Palaeolithic reference profiles based on the representation of adults and juveniles documented at Chagyrskaya^38,39^ (see Supplementary Data 6), for a total of five reference demographic models (Table 2, Supplementary Table S10). For each simulation, six individuals were randomly drawn (with and without replacement) to match the minimum number of individuals (MNI) represented in the Goyet assemblage. The frequencies with which the exact Goyet composition was replicated provided estimates of its probability under each demographic model^37–39^. As a comparative framework, we applied the same procedure to the cannibalised El Sidrón Neandertal assemblage^93^, providing a comparison with another cannibalised group; and to the Chagyrskaya assemblage, serving as a reference Neandertal population^38,39^ (Supplementary Data 6).

This approach allows us to assess whether the demographic profile of the Goyet assemblage is compatible with random selection in a theoretical reference sample, comparable to what could be expected in another Neandertal assemblage, or whether it instead reflects a selective bias distinct from theoretical expectations.

### Genetic sexing

A total of 16 remains from the Troisième caverne of Goyet were sampled in a dedicated clean room facility at the Royal Belgian Institute of Natural Sciences (Brussels, Belgium), except for two (Goyet 2878-2D and Q305-1) sampled at the University of Tübingen (Germany). We first removed ~ 1 mm of surface material using a sterile dentistry drill, and then drilled between 1 and 57.1 mg of skeletal powder. Whenever possible, multiple small sub-samples were taken from the same bone.

Shotgun sequencing was used to test for ancient DNA preservation (Supplementary Data 1). This revealed the presence of sufficient endogenous DNA (i.e. deamination frequencies at either end of the fragments above the standard 10% threshold) in 14 specimens. However, using AuthentiCT^94^, contamination estimates were observed to be above 10% for all samples except for one (Q305-1; Supplementary Table S3). Therefore, all downstream analyses were conducted using only deaminated sequences. Genetic sexing was obtained by comparing the sequence coverage of the X chromosome to that of the autosomes. Several of the specimens had too few sequences covering the X chromosome (< 100) to securely determine their sex (Supplementary Data 1).

For two of the specimens with deamination frequencies below 10% (2878-2D and D183-4), filtering of the shotgun sequences for deamination signal resulted in data loss that would not allow sex determination. As a result, nuclear DNA capture was performed for genetic libraries of the two elements^95^. Specimen 2878-2D did not show evidence of ancient DNA damage in the captured data and was excluded from further analysis. Instead, the captured data of specimen D183-4 showed low but detectable levels of damage (deamination frequency of 3.3% at the 5’ end). We then performed filtering of deaminated reads using PMDtools^96^ with increasing thresholds (Supplementary Data 1). The filtered data was then used for sex determination^97^.

### Carbon and nitrogen stable isotope analysis

We analysed the collagen of Goyet D183-4 and Q305-1 for *δ*^13^C and *δ*^15^N in order to compare them to the ratios already published for other Neandertals at Goyet^33^. Sample preparation followed Krajcarz et al.^98,99^ and isotopic measurements were performed as described in Wißing et al*.*^9^.

### Estimation of bone length and stature

The maximum length (MaxL) of the lower limb bones represents a valuable proxy to infer individual stature^100–102^, and the estimation of biomechanical length (BML) was required for cross-section positioning^103,104^. For incomplete specimens, femoral and tibial MaxL were obtained using Jacobs’s^105^ equations, while the BML was either derived from MaxL, scaled with a reference bone of known BML, or in the case of some Neandertals, using regression between BML and the distance between anatomical landmarks from several complete specimens (Supplementary Data 4). One of the studied Goyet bones (GN2-FemII) was not sufficiently preserved to securely assess its length. We did not estimate the length of GN-RadI either due to a lack of reference for Pleistocene samples. Instead, cross-sections were placed at the maximum extension of the radial interosseous crest since it has been shown to be a stable landmark in the radial diaphysis and has been used as a reference for cross-sectional analysis in Pleistocene hominins^106^.

Individual stature was estimated using the Sjøvold^43^ formulae, as previously done on other Pleistocene hominins^107^. Due to shorter limb distal segments in European Neandertals compared to both Holocene and Pleistocene *Homo sapiens* populations^108–110^, we prioritised stature estimations based on femoral MaxL when both femur and tibia were available for a single individual (Table 3). Differences in estimated stature between the Neandertals from Goyet and the Neandertal comparative sample were assessed using Welch’s t-test, which does not assume equal variances between groups, thus accounting for potential heteroscedasticity and unbalanced sample size in the comparative datasets^111^.

### Cross-sectional properties

Cross-sectional geometry (CSG) properties were calculated using the *morphomap* R package^12^ based on 3D reconstructions of CT and microCT scans. All bones were virtually aligned at their distal extremities following Ruff^103^ prior to CSG computation. To perform the alignment of incomplete bones, we used the best available preserved fossil specimen as a reference: Spy 6 for the radius, Spy 8 for the femur, and Spy 9 for the tibia. The reference specimen was scaled to correspond to the estimated BML of each target specimen and the target specimen was positioned using anatomical landmark alignment and mesh-surface match to the reference.

CSG properties include second moments of area Imax and Imin that provide indications on the cross-sectional shape and cortical distribution, and polar section modulus Zp, which is proportional to bending and torsional resistance^112^.

We analysed cross-sections at standard locations that were preserved on at least one Goyet specimen, but also commonly used in the literature in order to ensure broad comparative data^27,58,113–118^. Since most of the Goyet long bones present incomplete diaphyses, a majority of the specimens preserve available data for only one of the locations (Table 4). These include 35%, 50%, and 80% of the BML for the femur; 20%, 35%, and 50% for the tibia; and at the point of maximum medial projection of the interosseous crest for the radius^119^. In cases where anatomical features were too poorly preserved to assess femoral and tibial lengths to locate the cross-sections, we used cross-sectional locations following Trinkaus and Ruff^27^. For the femur, the cross-section at 80% was placed at the maximum extent of the gluteal buttress, and 65% at the point where the proximal muscle insertion lines converge into the linea aspera proper. Midshaft (50%) was located at the maximum extent of the pilaster and at the narrowest shaft breadth on early *Homo sapiens* femora. On the tibial diaphysis, midshaft (50%) is where the soleal line meets the posteromedial edge of the diaphysis while the mid-proximal section (65%) is close to the position of the nutrient foramen, especially on the tibial pilaster when one is present. The mid-distal (35%) cross-section approximates the distal minimum circumference and can be located as such.

Diaphyseal robusticity indices should be standardised by body size—calculated as the product of body mass and bone length—to allow meaningful comparisons, particularly for behavioural studies^29^. However, in the case of highly fragmented remains, such as the Goyet specimens, estimating body mass is not possible due to the absence of key skeletal elements, particularly the femoral head^120,121^. Given this limitation, diaphyseal robusticity indices were primarily used to reconstruct biological profiles rather than for direct behavioural inference.

### Cross-sectional shape

Commonly used CSG parameters do not precisely describe the diaphyseal cross-sectional shape or its variation in long bones^122,123^, despite cross-sectional shape being a reliable indicator of bending rigidity and, by extension, of behaviours that cause bending stresses^124^. Therefore, we used a 2D landmark-based geometric morphometric (GMM) approach to assess cross-sectional shapes accurately^64,125^. We performed these analyses at the same diaphyseal locations as the CSG. A total of 42 landmark coordinates (21 for endosteal and 21 for periosteal surfaces) were automatically extracted from each section using the *morphomap* R package^12^. We performed a Procrustes superimposition and a PCA analysis on the landmarks’ Procrustes coordinates in order to describe cross-sectional shape distinctions between Neandertals, UPHS and Neolithic samples (Table 1). GMM analysis was performed using the MorphoJ software^126^ and the *geomorph* R package^127^.

## Supplementary Information

Below is the link to the electronic supplementary material.

Supplementary Material 1

Supplementary Material 2

Supplementary Material 3

Supplementary Material 4

Supplementary Material 5

Supplementary Material 6

## Data Availability

Detailed information and data are presented in the online supplementary material of this article. The palaeogenomic data of this study have been deposited in the European Nucleotide Archive (ENA), under accession numbers PRJEB98924 (shotgun sequencing data) and PRJEB98734 (capture data).
