# Supplementary material for: Highly selective cannibalism in the Late Pleistocene of Northern Europe reveals Neandertals were targeted prey
Source: Sci Rep. 2025 Nov 19;15:40741. doi: 10.1038/s41598-025-24460-3 (PMC12630619; doi:10.1038/s41598-025-24460-3)
Supplement: Supplementary file 1 — Supplementary Material 1 [file 41598_2025_24460_MOESM1_ESM.docx]

**Supplementary Information**

**Neandertals as prey: highly selective cannibalism in the Late Pleistocene**

Quentin Cosnefroy, Isabelle Crevecoeur, Patrick Semal, Mateja Hajdinjak, Alba Bossoms Mesa, Johannes Krause, Guido Alberto Gnecchi-Ruscone, Cosimo Posth, Hervé Bocherens, Thibaut Devièse, Hélène Rougier

**Table of Contents**

[Supplementary Data 1. Genetic sex determination 4](#_Toc207973099)

[1.1. Shotgun sequencing 4](#_Toc207973100)

[1.2. Nuclear DNA capture 5](#_Toc207973101)

[1.3. Genetic sexing 6](#_Toc207973102)

[Supplementary Data 2. Stable isotope analysis 7](#_Toc207973103)

[Supplementary Data 3. The Neandertal upper and lower limb long bone remains from Goyet 8](#_Toc207973104)

[3.1. Humerus 8](#_Toc207973105)

[3.2. Radius 8](#_Toc207973106)

[3.3. Femur 9](#_Toc207973107)

[3.4. Tibia 11](#_Toc207973108)

[Supplementary Data 4. Bone length estimation 14](#_Toc207973109)

[4.1. Maximum length estimation 14](#_Toc207973110)

[4.2. Biomechanical length estimation 14](#_Toc207973111)

[4.3. Repeatability 15](#_Toc207973112)

[Supplementary Data 5. Neandertal sex determination in the absence of genetic data 17](#_Toc207973113)

[Supplementary Data 6. Random resampling procedures 19](#_Toc207973114)

[6.1. Theoretical models (Ledermann tables) 19](#_Toc207973115)

[6.2. Archaeological model (Chagyrskaya site) 19](#_Toc207973116)

[6.3. Resampling 19](#_Toc207973117)

[Supplementary Tables 21](#_Toc207973118)

[Supplementary Table S1. The Goyet Neandertal assemblage with the biological information and individual associations of the remains, and indication of anthropogenic modifications. 21](#_Toc207973119)

[Supplementary Table S2. Sample information and radiocarbon dating results of the Neandertals from Goyet. 24](#_Toc207973120)

[Supplementary Table S3. Summary of sequencing data of the Goyet libraries analysed in the present study. 25](#_Toc207973121)

[Supplementary Table S4. Sex determination of Goyet D183-4 using PMDtools-filtered nuclear capture data. 25](#_Toc207973122)

[Supplementary Table S5. Stable isotope values of the Neandertals from Goyet. 26](#_Toc207973123)

[Supplementary Table S6. Methods employed for estimating the maximum length (MaxL) and biomechanical length (BML) of long bones. 27](#_Toc207973124)

[Supplementary Table S7. Distance between anatomical landmarks of complete and subcomplete Neandertal femora. 30](#_Toc207973125)

[Supplementary Table S8. Detailed compositions of the comparative samples of radiuses, femurs, and tibias used in the cross-sectional geometry analyses. 31](#_Toc207973126)

[Supplementary Table S9. Stature estimation of the Neandertal sample. 31](#_Toc207973127)

[Supplementary Table S10. Demographic profiles of the reference populations. 31](#_Toc207973128)

[Supplementary Table S11. Intra- and inter-observer variation in bone length estimates using the scaling method and its impact on stature estimations. 32](#_Toc207973129)

[Supplementary Figures 33](#_Toc207973130)

[Supplementary Figure S1. Coverage ratio between the X chromosome and the autosomes. 33](#_Toc207973131)

[Supplementary Figure S2. Bivariate plot of the δ^15^N vs δ^13^C values of the collagen of the Neandertals from Goyet. 34](#_Toc207973132)

[Supplementary Figure S3. Radial cross-sectional shape analysis based on Procrustes coordinates from the endosteal and periosteal contours at the maximum medial extension of the interosseous crest. 35](#_Toc207973133)

[Supplementary Information References 36](#_Toc207973134)

# Supplementary Data 1. Genetic sex determination

## 1.1. Shotgun sequencing

*Shotgun data production.* Sixteen of the Neandertal remains from Goyet were sampled for genetic analysis. At the Max Planck Institutes for Evolutionary Anthropology in Leipzig and for the Science of Human History in Jena, we extracted and purified the DNA using specialised protocols for highly fragmented DNA, i.e. Rohland *et al.*^[1]^ for 87 extracts and Dabney *et al.*^[2]^ for three extracts (Supplementary Table S3). For one third of the extracts this step was performed manually, and for the remaining two thirds it was implemented on an automated Agilent Technologies Bravo NGS workstation B. We used between 5 μl to 30 μl of extract to produce 115 single-stranded DNA libraries^[3,4]^. Notably, 15 of these libraries were subjected to uracil–DNA–glycosylase (UDG) and Endonuclease VIII (Endo VIII) treatment (i.e. USER treatment), which removed uracils present in the middle of the sequences, while preserving uracils at their ends for ancient DNA authentication^[3]^. We quantified these libraries through quantitative PCR (qPCR) using a spiked control oligonucleotide for reference^[5]^. Each library was barcoded with a unique 7 or 8 base pair (bp) index to allow for multiplex sequencing^[6,7]^.

The shallow shotgun sequencing was performed on Illumina’s MiSeq and HiSeq platforms with double-index pair-end configurations. We used the software by Illumina (Bustard) to call the bases for these sequences. Then, we trimmed their adapters and merged them by the paired-ends that overlapped using *leeHom* (<https://grenaud.github.io/leeHom/>). As a result, we obtained an average of 2.85 million raw sequences per library.

We aligned these raw sequences to the hg19 (GRCh37) human reference genome^[8]^ using the Burrows-Wheeler Aligner version 0.5.10^[9]^ with the ancient parameters described in [Meyer *et al.*](https://www.zotero.org/google-docs/?PvD0qp)^[10]^. For quality-control purposes, we filtered the sequences for a minimum mapping quality of 25 and a minimum length of 35 bp. The number of unique mapped sequences varied largely between libraries, from 208 to 524,434 sequences.

*Shotgun data authentication and analysis.* For 31 of the libraries described above there was no evidence of authentic ancient DNA preservation, i.e. the deamination frequency at either of the ends of the sequences did not reach a minimum of 10%. While for most cases this could be overcome because at least one of the libraries from a given skeletal element was positive, this was not the case for Goyet 2878-2D and Goyet D183-4.

For the remaining 84 libraries that were positive for ancient DNA preservation, we estimated the present-day human DNA contamination levels using AuthentiCT^[11]^. In the large majority of cases contamination exceeded 10%, and only one library from Goyet Q305-1 had contamination estimates lower than 2%. In light of this, we restricted our analyses to deaminated sequences only, and merged the data from the libraries that originated from the same skeletal element.

## 1.2. Nuclear DNA capture

For the original non-UDG library of sample Goyet D183-4, and for a new half-UDG library^[12]^ of sample Goyet 2878-2D, we performed an in-solution targeted enrichment of 1.24M SNPs across the human genome^[13]^. While this approach is usually implemented for obtaining genomic data from ancient modern humans, the resulting genomic coverage can be used to perform sex determination for Neanderthal specimens as well. The enriched genetic libraries were sequenced on a HiSeq Illumina platform with a pair-end configuration for around 11M cluster (50+50 cycles) and 6M clusters (100+100 cycles), respectively. We processed the data through EAGER v1^[14]^ where reads where trimmed from adaptors, retained only merged reads using Clip&Merge, and mapped them to the hg19 reference genome using the Burrows-Wheeler Aligner^[9]^ with parameters -n 0.01-l 16500. We then filtered the sequences for a minimum length of 30 bp and a minimum mapping quality of 30 resulting in 128,362 (Goyet D183-4) and 99,708 (Goyet 2878-2D) unique mapped sequences. Damage pattern was calculated with mapDamage2.0^[15]^. Goyet 2878-2D resulted in ~1% of C to T substitutions at the 5’ end and in a mean fragment length of 85 bp, which is not consistent with the presence of ancient hominin DNA in the captured data and was thus excluded from genetic sexing. Instead, sample Goyet D183-4 resulted in 3.3% of C to T substitutions at the 5’ end and in a mean fragment length of 63 bp, and was used for sex determination on deaminated restricted data. We applied PMDtools^[16]^ with two different parameters, i.e. customterminus, which filters data with evidence of C to T substitutions at the 5’ and 3’ ends of the molecules (tested for 1, 2, 3 bp at both ends), and with a pmdscore, which probabilistically estimates the damage status of each read (tested for pmd scores from 3 to 10).

## 1.3. Genetic sexing

*Genetic sexing using shotgun data.* In order to infer the genetic sex of the analysed skeletal elements from Goyet, we compared the number of bases covered by ancient sequences on the X chromosome versus on the average of the autosomes, using samtools depth (<https://github.com/samtools/samtools>) and custom R scripts. For male individuals, we would expect this ratio to be approximately 0.5, and approximately 1 for female individuals. As a reference for these expectations, we used the published shotgun data of Spy 94a (a male Neandertal) and of Les Cottés Z4-1514 (a female Neandertal) (Supplementary Fig. S1)^[17]^. Taking these expectations into consideration, we concluded that Goyet Q56-1 (Femur I), Goyet Q57-1 (Tibia II), Goyet Q57-2 (Femur II), Goyet Q57-3 (Tibia III), Goyet Q55-4 (Tibia IV), Goyet Q374a-1 (Tibia V), Goyet Q305-7, and Goyet Q305-4 (Tibia I) are females, and Goyet Q305-1 is a male. The rest of the Goyet skeletal elements had less than 100 sequences on the X chromosome. Therefore, we were unable to sex them with the present data to the limits of our resolution.

*Genetic sexing using nuclear capture*. We performed sex determination with the PMDtools filtered reads of the nuclear captured data from Goyet D183-4 using the X-rate estimate approach published in Mittnik *et al.*^[18]^. The data is consistent with a male sex for all filtered datasets with increased stringency in the selection of deaminated reads (Supplementary Table S4).

# Supplementary Data 2. Stable isotope analysis

Eighteen Goyet Neandertal specimens had previously been analysed for the stable isotope values *δ*^13^C, *δ*^15^N, and *δ*^34^S of their collagen^[19,20]^ (Supplementary Table S5). All of them represent adult or adolescent individuals^[21]^. Their δ^15^N values range from 10.7‰ to 12.5‰ with an average of 11.6 ± 0.43‰, and their *δ*^13^C values from −19.7‰ to −19.0‰ with an average of −19.3 ± 0.22‰. Note that 2878-2D, which represents individual GN6, has the highest *δ*^15^N value of the assemblage whereas its *δ*^13^C value matches the ratios calculated for other specimens (Supplementary Fig. S2). This can be explained by the fact that 2878-2D is a tooth and *δ*^15^N values of dentin are generally higher than those of the bones of the same individuals^[22,23]^.

In the present study, two additional Neandertal specimens were sampled and their collagen analysed for carbon and nitrogen stable isotopic values: D183-4 and Q305-1 (Supplementary Table S1). With values of C_coll_/N_coll_ = 3.3 and 3.2, and N_coll_ = 14.6% and 14.0% for D183-4 and Q305-1, respectively, collagen preservation is within acceptable ranges for both specimens, i.e. C_coll_/N_coll_ atomic ratios are between 2.9 and 3.6 and nitrogen percentages of the collagen (N_coll_) are above 5%^[24,25]^. As such, the *δ*^13^C and *δ*^15^N values of D183-4 and Q305-1 can be compared to those of the other Goyet Neandertal specimens showing that they are within the variation of the group (Supplementary Table S5; Supplementary Fig. S2). As a result, the child represented by D183-4 (individual GN4?) may have belonged to the same group as the Neandertal adults/adolescents identified at Goyet, or to a different group that would have had the same diet as the adults/adolescents found at Goyet. The femoral diaphysis Q305-1 represents individual GN5. Its femoral length of 76 mm corresponds to a neonate (see Supplementary Data 3). If one of the female adult/adolescent individuals was his mother, his stable isotope values indicate that he died before maternal milk consumption would have started to change his *δ*13C and *δ*15N ratios compared to those of his mother.

# Supplementary Data 3. The Neandertal upper and lower limb long bone remains from Goyet

The preserved long bone remains from the upper limb (humerus and radius) and the lower limb (femur and tibia) of the Neandertals from Goyet are listed in Supplementary Table S1 and briefly described below. We used these descriptions to select the elements analysed in the present study.

## 3.1. Humerus

- Humerus II (Q376-20), 90 mm long fragment of a right humeral proximal diaphysis.

The fragment preserves the intertubercular sulcus and the distal part of the crest of the greater tubercle. Less than one-third of the diaphyseal circumference is preserved.

- Humerus III, made of two fragments (Q53-4, Q56-14) that preserve 89 mm of an incomplete right humeral proximal diaphysis and extremity.

The first fragment (Q53-4) preserves the crest of the lesser tubercle while Q56-14 preserves the medial part of the anatomical neck. In its most proximal part, the diaphysis preserves approximately two-thirds of the diaphyseal circumference.

- Q375-10, 37 mm long fragment of a right humeral diaphysis and anatomical neck.

Rougier *et al.*^21^ hypothesised that Q375-10 might belong to Humerus II based on the complementary portions preserved on both specimens, but no refit was found. It appears more parsimonious not to assign Q375-10 to a particular element given the number of individuals represented in the Neandertal sample and the low level of morphometric variability observed.

- Q376-35, 37.3 mm long fragment of a humeral head.

Most of the articular surface is preserved. No other remarkable feature is present.

## 3.2. Radius

- Radius I, made of four fragments (Q56-12, Q56-13, Q375-7, Q375-8) preserving 122 mm of a right radial diaphysis.

The preserved part extends from the infra-tuberosity region to the mid-distal diaphysis. The diaphyseal circumference is preserved on a length of 61 mm (taken parallel to the longitudinal axis of the bone).

- Q116-3, 26 mm long fragment of a radial head and neck.

A vertical fracture led to half of the radial head along with half of the neck down to the beginning of the radial tuberosity to be preserved. The preserved portion of the articular circumference has an even height.

## 3.3. Femur

- Femur I, made of four fragments (Q56-1, Q98-1, Q375-1, Q376-2) that preserve 162 mm of a right femoral diaphysis and partial proximal extremity.

The proximal extremity of the specimen preserves only its posteromedial aspect, with a fully preserved lesser trochanter and the lower portion of the neck. The subtrochanteric region preserves almost a complete diaphyseal circumference at the interface of the four fragments, which could be virtually reconstructed for the cross-sectional analysis. The distal portion of the specimen preserves the posterior and posterolateral surfaces of the diaphysis with the proximal part of the *linea aspera* and the nutrient foramen.

- Femur II, made of two fragments (Q56-7, Q57-2) that preserve 127 mm of a right femoral diaphysis.

The specimen preserves the midshaft and mid-distal parts of the diaphysis. A vertical fracture extends along the anteromedial surface, slightly affecting the diaphyseal circumference that was virtually reconstructed for cross-sectional analysis. The lateral side is the best preserved portion of the element.

- Femur III, made of three fragments (Q55-5, Q115-2, 1189-1) that preserve 142 mm of a left femoral diaphysis.

The specimen is preserved from the mid-distal to the distal diaphysis. The proximal end of Femur III preserves the entire diaphyseal circumference on approximately 15 mm, including the distal end of the *linea aspera* proper. The distal part of the specimen preserves only the anterolateral surface and part of the lateral supracondylar line. The posterior surface and the popliteal surface are not preserved in the distal extension of the specimen.

- Femur IV, made of two fragments (Q56-6, Q115-1) that preserve 177 mm of a right femoral diaphysis.

This is a long fragment of lateral surface of femoral diaphysis, from around mid-diaphysis down to its distal end. The specimen is regularly rounded antero-posteriorly but becomes flattened in its distal portion. Note that Femur IV had been erroneously assigned to the left side in Rougier *et al.*^[21]^. Its revised identification as a right bone means that it belongs to a different individual from the one represented by right Femur II because of overlapping preserved parts in the mid-diaphysis area. Although seemingly unlikely, we cannot entirely rule out that Femur IV belongs to the same element as right Femur I.

- Q55-7, 78 mm long fragment of a right femoral diaphysis.

Located in the distal half of the diaphysis, Q55-7 preserves the upper part of the popliteal surface and the medial supracondylar line on its whole length. The inferior section of the fragment is rounded, which is compatible with the absence of a pilaster on the element. The side of Q55-7 was not determined in Rougier *et al.*^[21]^. The detailed description of the fragment location that we propose here allows us to assign it to the right side. Based on the absence of overlapping preserved areas, we cannot exclude Q55-7 from belonging to right Femurs I, II or IV.

- Q305-1, 76 mm long fragment of a right femoral diaphysis.

Sub-complete right femoral diaphysis, with the proximal and distal metaphyseal ends of the element slightly eroded. The estimation of the diaphyseal length allows us to assign Q305-1 to a neonate (-0.14 +/- 0.279 years old) using Cowgill’s^[26]^ equation corrected for Neandertals, as well as when comparing it to other Holocene and Pleistocene samples^[26,27]^. Its robusticity compared to its size pointed to an archaic status, and the Neandertal status of the element was confirmed by ancient DNA analysis^[28]^

- Q305-11, 80 mm long fragment of a femoral diaphysis.

Convex portion of femoral diaphysis without remarkable features. Originally identified as a tibial diaphysis fragment^[21]^, Q305-11 is reassigned here to a femoral diaphysis because of the thickness of its cortical bone.

- Q305-12, 84 mm long fragment of a femoral diaphysis.

Thick fragment of medio-laterally or antero-posteriorly convex femoral diaphysis. The fragment does not preserve any remarkable features.

- Q375-9, 82 mm long fragment of a left femur diaphysis.

Probably located just below mid-diaphysis, the fragment mainly preserves the medial surface and is strongly convex between its anterior and posterior edges, the latter showing a very slight vertical depression marking the edge of the *linea aspera* area on the posterior surface. Q375-9 does not preserve other remarkable features and only half of the diaphyseal circumference. Given that left femur III preserves a more distal part of the diaphysis, we cannot exclude that Q375-9 belongs to the same element.

- Q115-3, 65 mm long fragment of a tibial or femoral diaphysis.

The fragment is rather flat. The thickness of the cortical bone and aspect of the spongy bone point to a tibia or femur fragment but the absence of remarkable features proscribes a reliable identification.

## 3.4. Tibia

- Tibia I, made of eight fragments (Q56-2, Q116-2, Q305-2, Q305-3, Q305-4, Q375-3, Q375-4, Q376-5) that preserve 174 mm of a left tibial diaphysis.

The specimen is preserved from the proximal end of the tibial tuberosity to the distal diaphysis. The anterior part is the best preserved and exhibits a complete anterior crest. The diaphyseal circumference is complete on almost 50 mm from the mid-distal to the distal shaft, and required only minor reconstruction for its cross-sectional analysis.

- Tibia II (Q57-1), 95 mm long fragment of a left tibial diaphysis.

The specimen preserves the anterior part of the proximal diaphysis. The tibial tuberosity is present at the proximal end and the upper fourth of the anterior crest is preserved below. Only a third of the diaphyseal circumference is preserved.

- Tibia III, made of four fragments (Q54-4, Q56-5, Q57-3, Q305-8) of a right tibial diaphysis.

This fragmentary right tibial diaphysis is made of a proximal posterior part (Q54-4 & Q56-5) and of a distal part (Q57-3 & Q305-8) that do not refit. The latter part was originally assigned to a different element (Tibia VI; Rougier *et al.*^21^). However, aDNA analyses have shown that Q54-4 and Q57-3 belong to the same individual^[29]^. Since both specimens are part of a tibia from the right side, they must belong to the same bone, Tibia III. Subsequently, element Tibia VI was eliminated from the inventory of Goyet human remains. The proximal part of Tibia III preserves 158 mm of the diaphyseal posterior surface and the distal part 102 mm of the distal diaphysis. Tibia III preserves at most only about a third of the diaphyseal circumference at its distal end.

- Tibia IV, made of six fragments (Q55-3, Q55-4, Q56-8, Q56-11, Q375-6, Q376-6) that preserve 201 mm of a right tibial diaphysis.

Tibia IV preserves the posterior face from the soleal line to mid-distal diaphysis. The lateral surface is better preserved than the medial one. The diaphyseal circumference is best preserved at midshaft and at the distal end of the specimen allowing for cross-sectional analyses after slight reconstruction.

- Tibia V, made of three fragments (Q56-9, Q56-10, Q374a-1) that preserve 168 mm of a right tibial diaphysis.

This specimen is preserved from the proximal end of the soleal line to the mid-distal region. The posterior face is the best preserved while the lateral aspect is missing on about two thirds of the length of the specimen. The diaphyseal circumference is fully preserved at the distal end of the specimen.

- Q54-5, 74 mm long fragment of a right tibial diaphysis.

The specimen preserves the anterior part of the diaphysis from the base of the tibial tuberosity to its proximal end. It was originally suggested that Q54-5 might belong to Tibia IV based on morphometrics and taphonomy^[21]^ but it could actually belong to any of the identified right tibias except for the one represented by Q376-18 (because of overlap between the preserved parts).

- Q305-7, 98 mm long fragment of a tibial diaphysis.

Long and narrow fragment of tibial diaphysis that was originally refit with Tibia III^[21]^. Its aDNA analysis has shown that it actually belongs to a different individual and is now assigned to Tibia V or its antimere^[29]^. The specimen does not preserve any remarkable features.

- Q375-2, 75 mm long fragment of a left tibial diaphysis.

Q375-2 preserves the proximal portion of the posterior face including the soleal line that runs along the lateral broken edge of the fragment. The specimen does not preserve the same area of the diaphysis as left Tibias I and II, hence it is not possible to exclude it from belonging to either of these elements.

- Q376-18, 128 mm long fragment of a right tibial diaphysis.

The specimen preserves the anterior portion of a right tibial diaphysis. It is long and narrow; and exhibits the anterior crest on its whole length. Based on morphometrics and taphonomy, Rougier *et al.*^[21]^ suggested that Q376-18 might belong to Tibia III. With the reassignment of the distal part of right tibial diaphysis Q57-3 & Q305-8 to Tibia III (see Tibia III above), it is not possible to assign Q376-18 to Tibia III anymore as they preserve distal parts that overlap. Q376-18 thus represents a right tibia other than Tibia III, IV or V.

- Q56-17, 58 mm long fragment of a tibial diaphysis.

The fragment does not preserve any remarkable features. We tentatively assign it to a tibial diaphysis based on morphometrics.

# Supplementary Data 4. Bone length estimation

## 4.1. Maximum length estimation

When a bone was fully preserved, the maximum length was directly measured on the virtual 3D reconstruction. For the femur, the maximum length refers to the distance between the most proximal point of the head to the most distal point of the medial condyle, measured parallel to the longitudinal axis of the diaphysis^[30]^. For the tibia, the maximum length refers to the distance between the most proximal point of the tibial plateau to the most distal point of the medial malleolus, measured parallel to the longitudinal axis of the diaphysis^[30]^. When a bone was incomplete and its maximum length could not be directly measured, we estimated it using its biomechanical length following Trinkaus and Ruff^[31]^ who determined relationships between different bone lengths (Supplementary Table S6). When the biomechanical length was not available either, we relied on Jacobs’s^[32]^ equations for both the femur and tibia to estimate their maximum lengths (see Supplementary Table S6).

## 4.2. Biomechanical length estimation

When available, the biomechanical length was directly measured on the virtual 3D reconstructions. For the femur, the biomechanical length refers to the distance between the most distal point of the neck and the midpoint of a tangent line passing distally through both condyles, measured parallel to the longitudinal axis of the diaphysis^[33]^. For the tibia, it refers to the distance between the most proximal point of the lateral tibial plateau and the center of the talocrural surface, measured parallel to the longitudinal axis of the diaphysis^[33]^. For fragmented bones, the biomechanical length was estimated based on the estimated maximum length derived from Jacobs’s^[32]^ equations, following the recommendations of Trinkaus and Ruff^[31]^ (see Supplementary Table S6). However, in some Neandertal specimens, preservation was insufficient to apply Jacobs’s^[32]^ method. In such cases, alternative reconstruction approaches were used to estimate biomechanical length. Details of these methods are presented below (see also Supplementary Table S6).

The biomechanical length of GN1-FemI was estimated by scaling and virtually fitting the Spy 8 specimen to align the base of the neck and the proximal end of the linea aspera proper of both bones. This distance is fairly consistent among eight Neandertal femora with known lengths, ranging from 18% to 22% of the femoral biomechanical length (R² = 0.22) (Supplementary Table S7). After scaling Spy 8 accordingly, its biomechanical length was measured to provide an estimated length for GN1-FemI.

The biomechanical length of GN-FemIII was estimated by measuring the distance (D) between the distal end of the linea aspera proper and the point where its lateral extension meets the lateral supracondylar line. This distance remains consistent among nine Neandertal femora with known lengths (R² = 0.7559; Supplementary Table S6). Given the high R² of this correlation, we directly applied the following equation to estimate the biomechanical length of GN-FemIII:

**FemBML = 1.5996D + 296.48 (R² = 0.7559)**

Due to the limited number of complete Neandertal tibiae available for identifying reliable measurements to infer tibial length, the Spy 9 specimen was used as a reference to estimate the lengths of GN2-TibIII and GN1-TibV. For GN2-TibIII, Spy 9 was scaled to fit the positions of the proximal beginning of the soleal line and of the nutrient foramen of the former bone. For GN1-TibV, the medial shift of the popliteal line and the distal diaphyseal circumference were used for scaling. Once Spy 9 was adjusted accordingly, its biomechanical length was measured and applied to estimate those of GN1-TibV and GN2-TibIII.

## 4.3. Repeatability

The scaling method used to estimate long bone lengths (based on Spy 8 for femora and Spy 9 for tibiae) was evaluated through reproducibility tests. Measurements were performed by three independent observers (QC, NM, and JH), as well as through three repeated trials by the same observer (QC, QC_bis, and QC_ter). The detailed results of the intra- and inter-observer tests are provided in Supplementary Table S11. The 95% confidence intervals for inter-observer comparisons of biomechanical length were 18.81 mm for Femur I, 17.67 mm for Tibia III, and 6.25 mm for Tibia V. The 95% confidence intervals for intra-observer comparisons were 7.99 mm, 7.28 mm, and 4.70 mm for the same bones, respectively. These differences lead to a maximum error of 5.3 cm in the estimated stature of individual GN1 (based on Femur I) and 5.8 cm in the estimated stature of GN2 (based on Tibia III).

# Supplementary Data 5. Neandertal sex determination in the absence of genetic data

In the absence of ancient DNA, determining the sex of Neandertal individuals based solely on skeletal features often leads to uncertain diagnoses^[34]^. A recent study applying methods with high levels of accuracy based on pelvic traits, originally developed for both extant and archaeological *Homo sapiens* populations^[35,36]^, has proposed a statistical diagnosis of known Neandertals^[37]^. However, the application of such methods can be limited due to the scarcity of well-preserved Neandertal pelvic bones and by the possibility that typical *Homo sapiens* patterns of dimorphism may not apply to hominins with large pelves like Neandertals^[38]^. When considering the limb bones, several features—including long bone lengths, humeral head diameters, and femoral shaft robusticity—have been suggested to be sexually dimorphic^[39]^ based on comparisons with the level of sexual dimorphism observed in *Homo sapiens* and hypothesised to be related to sexual division of labour^[40]^. If none of these traits can reasonably provide definitive diagnoses, the presence of two distinct morphologies among Neandertal postcranial remains does not allow to refute the hypothesis that Neandertals present limb bone dimorphism^[39]^. For instance, the Palomas 96 individual presents a female-like greater sciatic notch morphology^[41]^. This sex determination is further supported by the overall gracility of its skeleton. Similarly, the marked difference in robusticity between La Ferrassie 2 and the more complete La Ferrassie 1, commonly attributed to a male based on pelvic morphology^[37,42]^, has led to the interpretation of the gracile La Ferrassie 2 individual as female, based solely on limb bone characteristics.

Concerning the local Neandertals from Belgium used as a comparative sample for the study of the Goyet specimens, the Spy left femur Spy 16 and left tibia Spy 9 were initially attributed to male individuals by Fraipont and Lohest^[43]^ and Hrdlička^[44]^, mainly based on their supposed association to cranium Spy 10, whose cranial superstructures they described as more pronounced than those of the other cranium Spy 1. While Hrdlička^[44]^ attributed the complete right femur Spy 8 to the same male individual, Fraipont and Lohest^[43]^ assigned it to the other Neandertal adult, and thus to a female individual. Since then, various authors have alternately classified the Spy specimens, including the infra-cranial remains, as male or female depending on the comparative samples and criteria they used (see Genovés^[45]^ for a detailed review). More recently, Hambücken^[46]^ refrained from assigning a definitive sex to the lower limb bones of the Spy Neandertals based on their morphometric study. The current absence of secure associations between cranial and infra-cranial remains—and even among the infra-cranial long bones—represents a major limitation to determining the sex of the Spy specimens^[47]^. Finally, both the Spy 8 and 16 femora and the Spy 9 tibia along with the Fonds-de-Forêt 1 femur were assessed as males by Trinkaus^[39]^ based solely on bone length. However, in a later study by Trinkaus and Ruff^[48]^ focusing on their structural properties, no definitive sex attribution was proposed, thus reflecting continued uncertainty in sexing infra-cranial remains in Neandertals.

Based on this literature review, we will consider Palomas 96 and La Ferrassie 2 as presumed females when assessing issues of sexual dimorphism and interpreting the biological variability of the Neandertals from Goyet, and we will refer to the local Neandertal specimens from Spy and Fonds-de-Forêt as undetermined.

# Supplementary Data 6. Random resampling procedures

To test whether the demographic profile observed at Goyet could have arisen by chance (i.e. could correspond to random sampling of a set of individuals from a population whose composition follows theoretical mortality profiles), we constructed demographic reference models based on both theoretical and archaeological mortality profiles. Each reference was modelled as an assemblage of 60 individuals since genomic evidence has suggested that it likely represents the upper limit of Neandertal community size^[49–53]^. Using this upper limit provides a conservative framework for the resampling process. The exact composition of each reference assemblage is presented in Supplementary Table S10.

## **6.1. Theoretical models (Ledermann tables)**

We used mortality profiles derived from Ledermann’s mortality tables (see tables from the *réseau 100, e_0_*)^[54]^, which provide age and sex distributions at death according to life expectancies at birth. We selected models with life expectancies at birth of 25, 30, and 35 years since they represent probable life expectancies in archaeological populations^[55–57]^.

## 6.2. Archaeological model (Chagyrskaya site)

The Chagyrskaya site benefits from extensive anthropological and palaeogenomic data providing minimum numbers of individuals and individual genetic identification and sexing^[51,53]^. The sample includes 11 or 12 individuals depending on whether Chagyrskaya D and E represent one or two distinct individuals^[53]^. Both compositions were used to ensure a conservative approach. The archaeological mortality profiles we computed reflect the individual representation pattern of the Chagyrskaya Neandertal assemblage applied to a population of 60 individuals (Supplementary Table S10).

## 6.3. Resampling

For each reference assemblage, we performed 10,000 random draws using the Excel Macro: *Resampling Stats for Excel*. Individuals were assigned to one of three categories: adult/adolescent females, adult/adolescent males, and juveniles (<15 years). Two different approaches were taken for the random draws:

- Without replacement: each draw removed the selected individual from the pool of individuals, simulating a scenario in which all Goyet individuals belonged to a single population.
- With replacement: individuals were returned to the pool after each draw, simulating the case in which the Goyet individuals originated from different groups. This scenario is considered less likely because stable isotopic analyses showed that the Goyet individuals share common dietary and geographical signals^[20]^, supporting their attribution to a single population.

For each run, we sampled six individuals to mirror the Goyet minimum number of individuals and we recorded the frequencies with which the exact composition of the Goyet sample (4 adult/adolescent females, 0 adult/adolescent males, 2 juveniles) was replicated across the 10,000 simulations.

In addition, we applied the same procedure to the El Sidrón cannibalised assemblage, which includes a minimum of 13 individuals whose sex has been estimated^[58]^. As one of the adults remains of unknown sex (Adult 7), we tested the El Sidrón assemblage under two alternative scenarios in which Adult 7 is either a male or a female. Finally, we applied the same procedure to the Chagyrskaya Neandertal assemblage (only in reference to the theoretical models), which comprises either 11 or 12 individuals depending on whether Chagyrskaya D and E? represent the same individual^[53]^; both scenarios were therefore tested.

# Supplementary Tables

## **Supplementary Table S1. The Goyet Neandertal assemblage with the biological information and individual associations of the remains, and indication of anthropogenic modifications.**

Data come from Rougier *et al.*^[21]^, Hajdinjak *et al.*^[17]^, and Bossoms Mesa *et al.*^[29]^, except when written in italics: present study. Rows with a gray filling indicate the elements analysed in the present study.

| **Bone piece** | **Identification** | **Age** | **Goyet Neandertal individual** | **Sex** | **Anthropogenic modifications** | | | |
| --- | --- | --- | --- | --- | --- | --- | --- | --- |
|  |  |  |  |  | **Cutmarks** | **Percussion notches** | **Percussion pits** | **Retoucher** |
| **Craniofacial skeleton** | | | | | | | | |
| 2878-3 + C5-1 | Right and left parietal fragments with portion of sagittal suture | Adu/ado |  |  |  |  |  |  |
| 2878-1 | Right parietal, postero-superior frag. | Adu/ado |  |  |  |  |  |  |
| 2878-2 | Right parietal, anterior frag. | Adu/ado |  |  |  |  |  |  |
| 2878-4 | Occipital, left nuchal plane frag. | Adu/ado |  |  |  |  |  |  |
| C5-6 | Right temporal, squamous frag. and complete petrous | Adu/ado |  |  | X? |  |  |  |
| C5-7 | Left temporal, mastoid portion frag. | Adu/ado |  |  |  |  |  |  |
| C5-8 | Left zygomatic, frontal process | Adu/ado |  |  |  |  |  |  |
| 2861-1 + 2878-1D | Right and left maxillae, alveolar and palatine processes with 9 teeth/dental roots + left I2 (isolated) | Adu/ado |  |  |  |  |  |  |
| 2878-8 + 2878-2D | Mandible, left body frag. with P1 and M1 + left P2 (isolated) | Adu/ado |  |  |  |  |  |  |
| C5-3 + C5-4 | Mandible, right gonial angle & ascending ramus frag. | Adu/ado |  |  | X |  |  |  |
| C5-5 | Mandible, left body inferior frag. | Adu/ado |  |  |  |  |  |  |
| 1424-3D* | Lower left I2 | Child | GN4 |  |  |  |  |  |
| **Trunk** | | | | | | | | |
| C5-2 | Lumbar vertebra 1-4, left pedicle and left superior articular process | Adu/ado |  |  |  |  |  |  |
| Q100-3 | Right rib 1, distal shaft frag. | Adu/ado |  |  |  |  |  |  |
| Q376-8 | Left rib 1, sub-complete | Adu/ado |  |  | X |  |  |  |
| Q376-13 | Right rib 2 shaft frag. | Adu/ado |  |  |  |  |  |  |
| Q376-16 | Right rib 3? shaft frag. | Adu/ado |  |  |  |  |  |  |
| Q376-32 | Left rib 3, frag. preserving the neck, costal tubercle and angle | Adu/ado |  |  |  |  |  |  |
| Q376-27 | Right? rib 3-7? shaft frag. | Adu/ado |  |  |  |  |  |  |
| Q376-29 | Right rib 3-9 shaft frag. | Adu/ado |  |  |  |  |  |  |
| Q376-30 | Right? rib 6-8?, sternal end | Adu/ado |  |  |  |  |  |  |
| Q376-9 | Right rib 11? shaft frag. | Adu/ado |  |  |  |  |  |  |
| Q55-6 + Q376-25 | Right rib 11? shaft | Adu/ado |  |  | X |  |  |  |
| Q376-26 | Rib 3-11 shaft frag. | Adu/ado |  |  |  |  |  |  |
| Q376-12 | Left rib 4?, shaft frag. with costal angle | Adu/ado |  |  |  |  |  |  |
| Q376-14 | Left rib 4-5?, distal half/third | Adu/ado |  |  |  |  |  |  |
| Q376-17 | Left? rib 4-9? shaft frag. | Adu/ado |  |  |  |  |  |  |
| Q376-28 | Left rib 5-9 shaft frag. | Adu/ado |  |  |  |  |  |  |
| Q119-2 | Left rib 7? shaft frag. | Adu/ado |  |  |  |  |  |  |
| Q376-7 | Left rib 7? shaft frag. | Adu/ado |  |  |  |  |  |  |
| Q376-11 | Left rib 10 shaft frag. | Adu/ado |  |  | X |  |  |  |
| Q376-31 | Left? rib 11?, distal half | Adu/ado |  |  |  |  |  |  |
| Q376-33 | Rib 8-11? shaft frag. | Adu/ado |  |  |  |  |  |  |
| **Upper limb** | | | | | | | | |
| Q55-1 | Left clavicle, lateral half | Adu/ado |  |  | X |  |  |  |
| *D183-4* | *Left clavicle, lateral 3/4* | *Child* | *GN4?* | *M* | *X* |  |  |  |
| Humerus II (Q376-20) | Right humerus diaphysis frag. | Adu/ado |  |  | X |  |  |  |
| Humerus III (2 spec.) | Right humerus, diaphysis and neck frag. | Adu/ado |  |  |  |  |  |  |
| Q375-10 | Right humerus, diaphysis and neck frag. | Adu/ado |  |  |  |  |  |  |
| Q376-35 | Humerus head frag. | Adu/ado |  |  |  |  |  |  |
| Radius I (4 spec.) | Right radius diaphysis | Adu/ado |  |  |  | X | X |  |
| Q116-3 | Radius, head and neck frag. | Adu/ado |  |  | X |  |  |  |
| Q53-5 | Ulna? diaphysis frag. | Adu/ado |  |  |  |  |  |  |
| 2878-37 | Hand middle phalanx 2-4 | Adu/ado |  |  |  |  |  |  |
| 2878-38 | Hand proximal phalanx 3-4, proximal extremity broken off | Adu/ado |  |  |  |  |  |  |
| 2878-39 | Left hand proximal phalanx 5, prox. extremity broken off, distal extremity partially broken | Adu/ado |  |  |  |  |  |  |
| Q376-1 | Hand proximal phalanx 2-4, both extremities broken off | Adu/ado |  |  |  |  |  |  |
| **Lower limb** | | | | | | | | |
| Femur I (4 spec.) | Right femur diaphysis & prox. extremity frag. | Adu/ado | GN1 | F | X | X | X |  |
| Tibia V (3 spec.) | Right tibia diaphysis frag. | Adu/ado | GN1 | *F* | X | X | X | X |
| Q305-7 | Tibia diaphysis frag. | Adu/ado | GN1 | *F* | X |  |  |  |
| Femur II (2 spec.) | Right femur diaphysis frag. | Adu/ado | GN2 | *F* | X | X | X |  |
| Tibia II (Q57-1) | Left tibia diaphysis frag. | Adu/ado | GN2 | *F* | X | X |  |  |
| Tibia III (*4 spec.*) | Right tibia diaphysis frag. | Adu/ado | GN2 | *F* | X | X | X | X |
| Tibia I (8 spec.) | Left tibia diaphysis | Adu/ado | GN3 | *F* | X | X |  |  |
| Tibia IV (6 spec.) | Right tibia diaphysis | Adu/ado | *GN6* | *F* | X | X | X | X |
| Q48-1 + Q376-36 | Left pubis | Adu/ado |  |  | X |  |  |  |
| Femur III (3 spec.) | Left femur diaphysis frag. | Adu/ado |  |  | X | X | X | X |
| Femur IV (2 spec.) | *Right* femur diaphysis frag. | Adu/ado |  |  | X | X | X |  |
| Q55-7 | *Right* femur diaphysis frag. | Adu/ado |  |  |  |  | X |  |
| *Q305-1* | *Right femur diaphysis* | *Neonate* | *GN5* | *M* |  |  |  |  |
| Q305-11 | *Femur* diaphysis frag. | Adu/ado |  |  |  |  |  |  |
| Q305-12 | Femur diaphysis frag. | Adu/ado |  |  |  |  |  |  |
| Q375-9 | *Left* femur diaphysis frag. | Adu/ado |  |  |  | X |  |  |
| Q115-3 | Tibia or femur diaphysis frag. | Adu/ado |  |  |  |  |  |  |
| Q54-5 | Right tibia diaphysis frag. | Adu/ado |  |  |  |  |  |  |
| Q375-2 | Left tibia diaphysis frag. | Adu/ado |  |  | X |  |  |  |
| Q376-18 | Right tibia diaphysis frag. | Adu/ado |  |  | X |  |  |  |
| Q56-17 | Tibia? diaphysis frag. | Adu/ado |  |  |  |  |  |  |

* Element numbers (Roman numerals) were given to the most complete bones of the Goyet human collection and indicate refits. Age: Adu/ado = adult or adolescent; Sex: M = male, F = female. All of the specimens are part of the Royal Belgian Institute of Natural Sciences collections and were excavated by E. Dupont in 1868, except for * that belongs to the Royal Museums of Art and History material from the early 20th century excavations of A. de Loë.

## **Supplementary Table S2. Sample information and radiocarbon dating results of the Neandertals from Goyet.**

| **ID** | **Goyet Neandertal Individual** | Radiocarbon dating | | |
| --- | --- | --- | --- | --- |
|  |  | Lab #* | ^14^C age (BP)* | Calibrated age (calBP) 95% probability** |
| 2878-2D |  | GrA-54028 | 32,190 +200/-190 | 36,970 - 36,160 |
| Q55-1 |  | GrA-54257 | 37,860 +350/-310 | 42,510 - 41,950 |
| *D183-4* | *GN4?* | *GrM13123* | *33,150 ± 220* | *38,880 - 37,030* |
| Humerus II (Q376-20) |  | GrA-60018 | 37,250 +320/-280 | 42,280 - 41,530 |
| Humerus III (Q53-4) |  | GrA-54022 | 39,870 +400/-350 | 44,030 - 42,670 |
| Q376-1 |  | GrA-46178 | 39,140 +390/-340 | 43,100 - 42,390 |
| Femur I (Q56-1) | GN1 | GrA-46170 | 38,440 +340/-300 | 42,740 - 42,180 |
| Femur II (Q57-2) | GN2 | GrA-54024 | 36,590 +300/-270 | 42,000 - 41,140 |
| Tibia II (Q57-1) | GN2 | GrA-46173 | 41,200 +500/-410 | 44,880 - 43,250 |
| Tibia III (Q57-3) | GN2 | GrA-60019 | 38,260 +350/-310 | 42,670 - 42,110 |
| Tibia I (Q305-4) | GN3 | GrA-46176 | 40,690 +480/-400 | 44,490 - 43,040 |
| *Q305-1* | *GN5* | *GrA-62471* | *32,650 ± 210* | *37,560 - 36,430* |

*Results published in Rougier *et al.*^[21]^ except for those written in italics published in Fotiadou *et al.*^[26]^. As noted in these publications, 2878-2D may have been varnished and its age should be considered to be underestimated, and the ages of D183-4 and Q305-1 also appear to be too young.

**Calibration of the radiocarbon dates was performed using the presently recommended calibration curve IntCal20^[59]^ and the OxCal software (version 4.4)^[60]^.

## **Supplementary Table S3. Summary of sequencing data of the Goyet libraries analysed in the present study.**

*Supplementary Table S3 is provided as supplementary material (Excel file).*

## **Supplementary Table S4. Sex determination of Goyet D183-4 using PMDtools-filtered nuclear capture data.**

*Supplementary Table S4 is provided as supplementary material (Excel file).*

## **Supplementary Table S5. Stable isotope values of the Neandertals from Goyet.**

| **ID** | **Element** | **Goyet Neandertal Individual** | ***δ* ^13^C (‰)** | ***δ* ^15^N (‰)** | ***δ* ^34^S (‰)** |
| --- | --- | --- | --- | --- | --- |
| C5-1 | Left parietal frag. |  | -19.7 | 12.1 | 10.3 |
| 2878-2D | Lower left P2 |  | -19.0 | 12.5 |  |
| Q376-9 | Right rib frag. |  | -19.2 | 11.8 | 12.9 |
| Q376-25 | Right rib frag. |  | -19.0 | 11.5 | 11.4 |
| Q119-2 | Left rib frag. |  | -19.3 | 11.5 | 11.9 |
| Q55-1 | Left clavicle frag. |  | -19.2 | 11.3 | 9.8 |
| *D183-4* | *Left clavicle frag.* | *GN4?* | *-19.5* | *11.3* |  |
| Humerus II (Q376-20) | Right humerus frag. |  | -19.4 | 11.8 | 11.6 |
| Humerus III (Q53-4) | Right humerus frag. |  | -19.0 | 11.7 | 9.7 |
| Q376-1 | Hand PP 2-4 |  | -19.2 | 10.9 |  |
| Femur I (Q56-1) | Right femur frag. | GN1 | -19.5 | 11.5 | 9.2 |
| Tibia V (Q374a-1) | Right tibia frag. | GN1 | -19.1 | 11.8 | 10.2 |
| Q305-7 | Tibia frag. | GN1 | -19.0 | 11.3 | 11.3 |
| Femur II (Q57-2) | Right femur frag. | GN2 | -19.1 | 11.9 | 10.8 |
| Tibia II (Q57-1) | Left tibia frag. | GN2 | -19.2 | 11.8 | 10.9 |
| Tibia III (Q57-3) | Right tibia frag. | GN2 | -19.6 | 11.2 | 10.9 |
| Tibia I (Q305-4) | Left tibia frag. | GN3 | -19.4 | 10.7 | 7.5 |
| Tibia IV (Q55-4) | Right tibia frag. | *GN6* | -19.2 | 11.6 | 11.4 |
| Q48-1 | Lt pubis |  | -19.6 | 11.3 | 11.5 |
| *Q305-1* | *Right femur* | *GN5* | *-19.3* | *10.9* |  |

All isotopic data and Goyet Neandertal individual attributions are from Wißing *et al.*^[19,20]^ and Bossoms Mesa *et al.*^[29]^, respectively, except for those in italics that are produced/proposed in the present study. The standards for isotopic data are the internationally defined marine carbonate V-PDB for *δ*^13^C, the standard atmospheric nitrogen (AIR) for *δ*^15^N and the internationally defined Vienna-Canyon Diablo Troilite (VCDT) for *δ*^34^S.

## **Supplementary Table S6. Methods employed for estimating the maximum length (MaxL) and biomechanical length (BML) of long bones.**

See Supplementary Data 4 for additional explanations.

| **Specimen** | **Side** | **MaxL (mm)** | **Method/reference** | **BML (mm)** | **Method/reference** |
| --- | --- | --- | --- | --- | --- |
| GN-RadI |  | ND | ND | ND | ND |
| GN1-FemI | Right | 408 | using biomechanical length^†^ | 383 | scaled with Spy 8 |
| GN2-FemII | Right | ND | ND | ND | ND |
| GN-FemIII | Left | 398 | using biomechanical length^†^ | 373 | using distance between the distal end of the *linea aspera* proper and the point where the lateral extension of the *linea aspera* meets the lateral supracondylar line |
| Burnot 3 | Right | 434 | using Jacobs (1992) F2+F3+F4 regression for unknown sex^‡^ | 407 | using maximum length following Trinkaus and Ruff^†^ |
| Burnot 4 | Left | 433 | using Jacobs (1992) F2+F3+F4 regression for unknown sex^‡^ | 407 | using maximum length following Trinkaus and Ruff^†^ |
| Burnot 7 | Right | 380 | using Jacobs (1992) F2+F3+F4 regression for unknown sex^‡^ | 356 | using maximum length following Trinkaus and Ruff^†^ |
| Hastière-Caverne B 36 | Right | 381 | using biomechanical length^†^ | 357 | direct measurement |
| Hastière-Caverne B 38 | Right | 398 | using biomechanical length^†^ | 373 | direct measurement |
| Hastière-CaverneM-54 | Left | 438 | using biomechanical length^†^ | 412 | direct measurement |
| Hastière-Petite Caverne 1 | Right | 423 | using biomechanical length^†^ | 397 | direct measurement |
| Hastière-Trou Garcon 22 | Left | 403 | using biomechanical length^†^ | 378 | direct measurement |
| Hastière-Trou Garcon 25 | Right | 438 | using biomechanical length^†^ | 412 | direct measurement |
| Sclaigneaux 144-2a | Right | 421 | using biomechanical length^†^ | 395 | direct measurement |
| Sclaigneaux 144-3 | Right | 492 | using Jacobs (1992) F2+F3+F4 regression for unknown sex^‡^ | 463 | using maximum length following Trinkaus and Ruff^†^ |
| Sclaigneaux 146-1 | Left | 437 | using biomechanical length^†^ | 410 | direct measurement |
| Sclaigneaux 147-6 | Left | 429 | using Jacobs (1992) F2+F3 regression for unknown sex^‡^ | 403 | using maximum length following Trinkaus and Ruff^†^ |
| GN3-TibI | Left | 292 | using Jacobs (1992) T2+T3 regression for females^‡^ | 271 | using maximum length following Trinkaus and Ruff^†^ |
| GN2-TibIII | Right | 324 | using biomechanical length^†^ | 303 | virtually scaled with Spy 9 using the proximal start of the soleal line and the nutrient foramen as anatomical landmarks |
| GN7-TibIV | Right | 313 | using Jacobs (1992) T3 regression for females^‡^ | 291 | using maximum length following Trinkaus and Ruff^†^ |
| GN1-TibV | Right | 306 | using biomechanical length^†^ | 282 | virtually scaled with Spy 9 using the medial shift of the popliteal line and distal diaphysis circumference as landmarks |
| Furfooz 23 | Right | 339 | direct measurement | 317 | direct measurement |
| Furfooz 30 | Left | 338 | direct measurement | 316 | direct measurement |
| Humain 7-11 | Left | 368 | direct measurement | 342 | direct measurement |
| Sclaigneaux 157-5 | Left | 364 | using Jacobs (1992) T3+T4 regression for unknown sex^‡^ | 341 | using maximum length following Trinkaus and Ruff^†^ |
| Sclaigneaux 165 | Left | 338 | direct measurement | 316 | direct measurement |
| Hastière A30 | Left | 336 | direct measurement | 313 | direct measurement |
| Hastière 37 | Left | 317 | direct measurement | 296 | direct measurement |
| Hastière 34 | Right | 288 | direct measurement | 267 | direct measurement |
| Hastière 55 | Left | 335 | direct measurement | 312 | direct measurement |

*^†^Maximum and biomechanical length relationships, from Trinkaus and Ruff*^[31]^

*Fem BML* *= FemMaxL x 0.949 – 4*

*Fem MaxL=FemBML x 1,037 + 11.2*

*Tib MaxL = 1.027 x TibBML + 13.7*

*Tib BML* *= (TibMaxL + 13.7)/1.027*

*^‡^Maximum length estimation from Jacobs*^[32]^

*F2+F3+F4 regression for unknown sex: Fem MaxL = 1.17F2+1.12F3+1.39F4+6.4 (R² = 0.979)*

*F2+F3 regression for unknown sex: Fem MaxL = 1.31F2+1.18F3+23.8 (R² = 0.962)*

*T2+T3 regression for females: Tib MaxL = 1.66T2+1.09T3+66.4 (R² = 0.837)*

*T3 regression for females: Tib MaxL = 0.84T3+221.7 (R² = 0.582)*

*T3+T4 regression for unknown sex: Tib MaxL = 0.96T3+1.25T4+98.8 (R² = 0.914)*

## **Supplementary Table S7. Distance between anatomical landmarks of complete and subcomplete Neandertal femora.**

The femoral biomechanical lengths are from Trinkaus and Ruff^[31]^*.*

| **Specimen** | **Fem BML** | **Neck-to-*linea aspera* length (mm)** | **Ratio of neck-to-*linea aspera* length to Fem BML (%)** | ***Linea aspera* proper-to-lateral supracondylar line length (mm)** | **Ratio of *linea aspera* proper-to-lateral supracondylar line length to Fem BML (%)** |
| --- | --- | --- | --- | --- | --- |
| Amud 1 | 458 | 83.49 | 18.23 | 90.08 | 19.69 |
| Fonds-de-Forêt 1 | 438 | ND | ND | 75.13 | 17.15 |
| La Chapelle-aux-Saints 1 (R) | 420 | ND | ND | 78.26 | 18.63 |
| La Chapelle-aux-Saints 1 (L) | 420 | 82.44 | 19.63 | 84.01 | 20.00 |
| La Ferrassie 1 (R) | 451 | 98.76 | 21.90 | 94.41 | 20.93 |
| La Ferrassie 1 (L) | 451 | 98.79 | 21.90 | 102.3 | 22.68 |
| La Ferrassie 2 (R) | 386 | 77.23 | 20.01 | 52.84 | 13.69 |
| La Ferrassie 2 (L) | 386 | 87.79 | 22.74 | 68.63 | 17.78 |
| Spy 8 | 411 | 97.62 | 23.75 | 74.94 | 18.23 |
| Spy 16 | 411 | 78.5 | 19.10 | ND | ND |

## **Supplementary Table S8. Detailed compositions of the comparative samples of radiuses, femurs, and tibias used in the cross-sectional geometry analyses.**

*Supplementary Table S8 is provided as supplementary material (Excel file).*

## **Supplementary Table S9. Stature estimation of the Neandertal sample.**

*Supplementary Table S9 is provided as supplementary material (Excel file).*

## **Supplementary Table S10. Demographic profiles of the reference populations.**

| **Site/model** | **N of individuals** | **N of female adults/adolescents** | **N of male adults/adolescents** | **N of juveniles (<15 years old)** |
| --- | --- | --- | --- | --- |
| Ledermann Q25 | 60 | 11 | 11 | 38 |
| Ledermann Q30 | 60 | 15 | 14 | 31 |
| Ledermann Q35 | 60 | 18 | 17 | 25 |
| Chagyrskaya^a^ | 60 | 16 | 16 | 28 |
| Chagyrskaya^b^ | 60 | 15 | 20 | 25 |

Qxx indicates the life expectancy at birth for each of Ledermann’s^[54]^ models used. For Chagyrskaya, the frequencies of female adults/adolescents (F), male adults/adolescents (M), and juveniles (JUV) were computed from the composition of the retrieved sample as outlined in Skov *et al.*^[53]^. Two different minimum numbers of individuals (MNI) were used (see Supplementary Data 6):

^a^MNI of 11: 3F/3M/5JUV

^b^MNI of 12: 3F/4M/5JUV

## **Supplementary Table S11. Intra- and inter-observer variation in bone length estimates using the scaling method and its impact on stature estimations.**

*Supplementary Table S11 is provided as supplementary material (Excel file).*

# Supplementary Figures


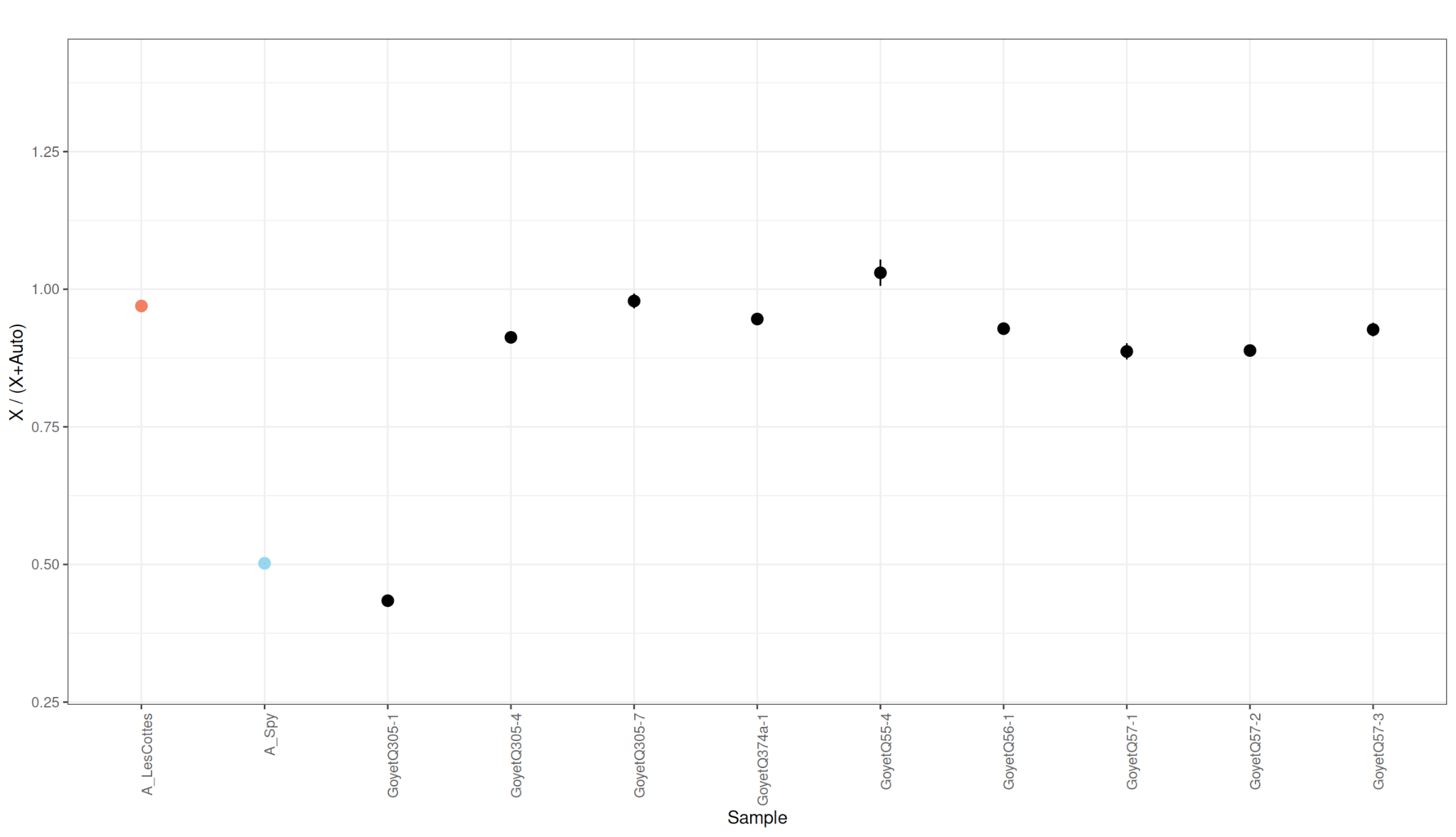


## **Supplementary Figure S1.** **Coverage ratio between the X chromosome and the autosomes.**

Data with 95% binomial confidence intervals for the Goyet Neandertal specimens as well as Spy 94a (male Neandertal reference) and Les Cottés Z4-1514 (female Neandertal reference)*.*


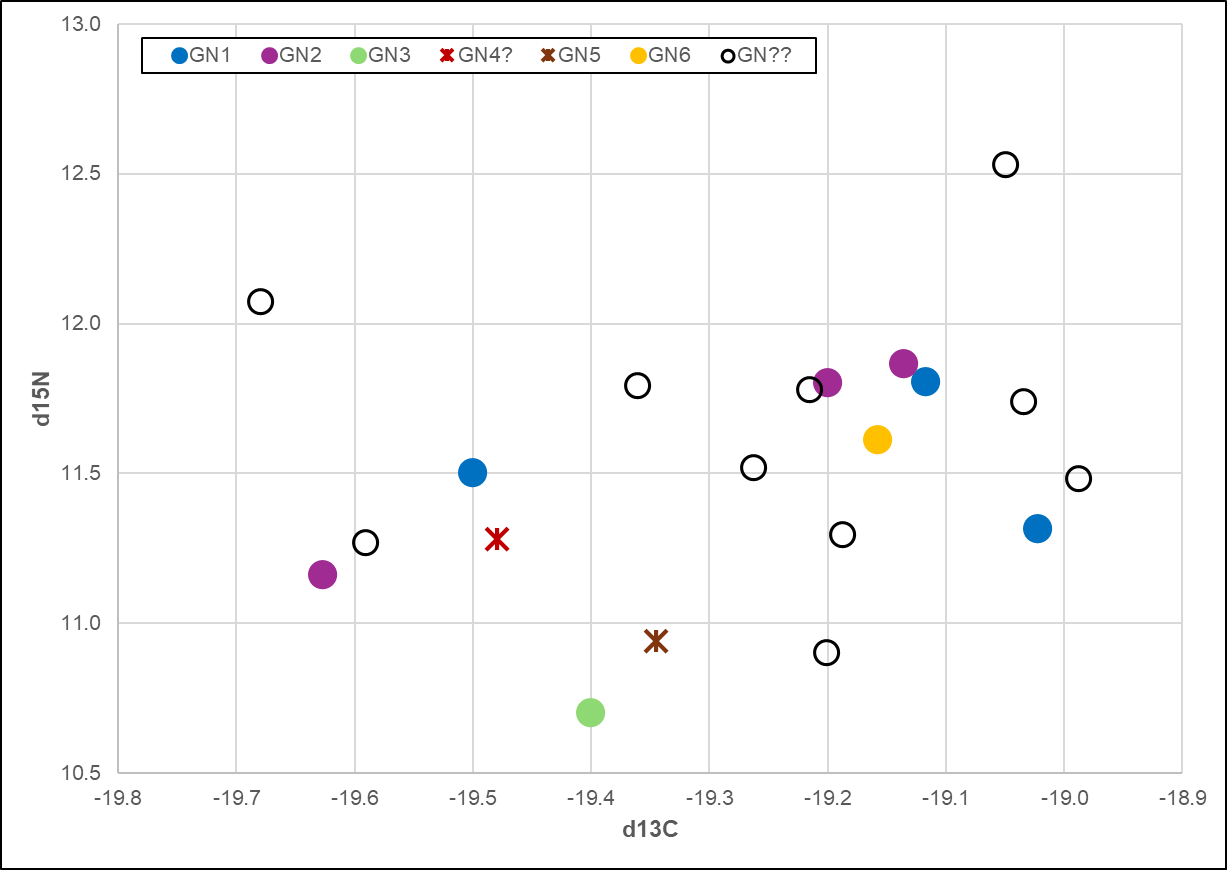


## **Supplementary Figure S2. Bivariate plot of** **the δ^15^N vs δ^13^C values of the collagen of the Neandertals from Goyet.**

Data are provided in Supplementary Table S5. Circles represent adult/adolescent individuals; crosses represent juvenile individuals. GN = Goyet Neandertal individual*.*


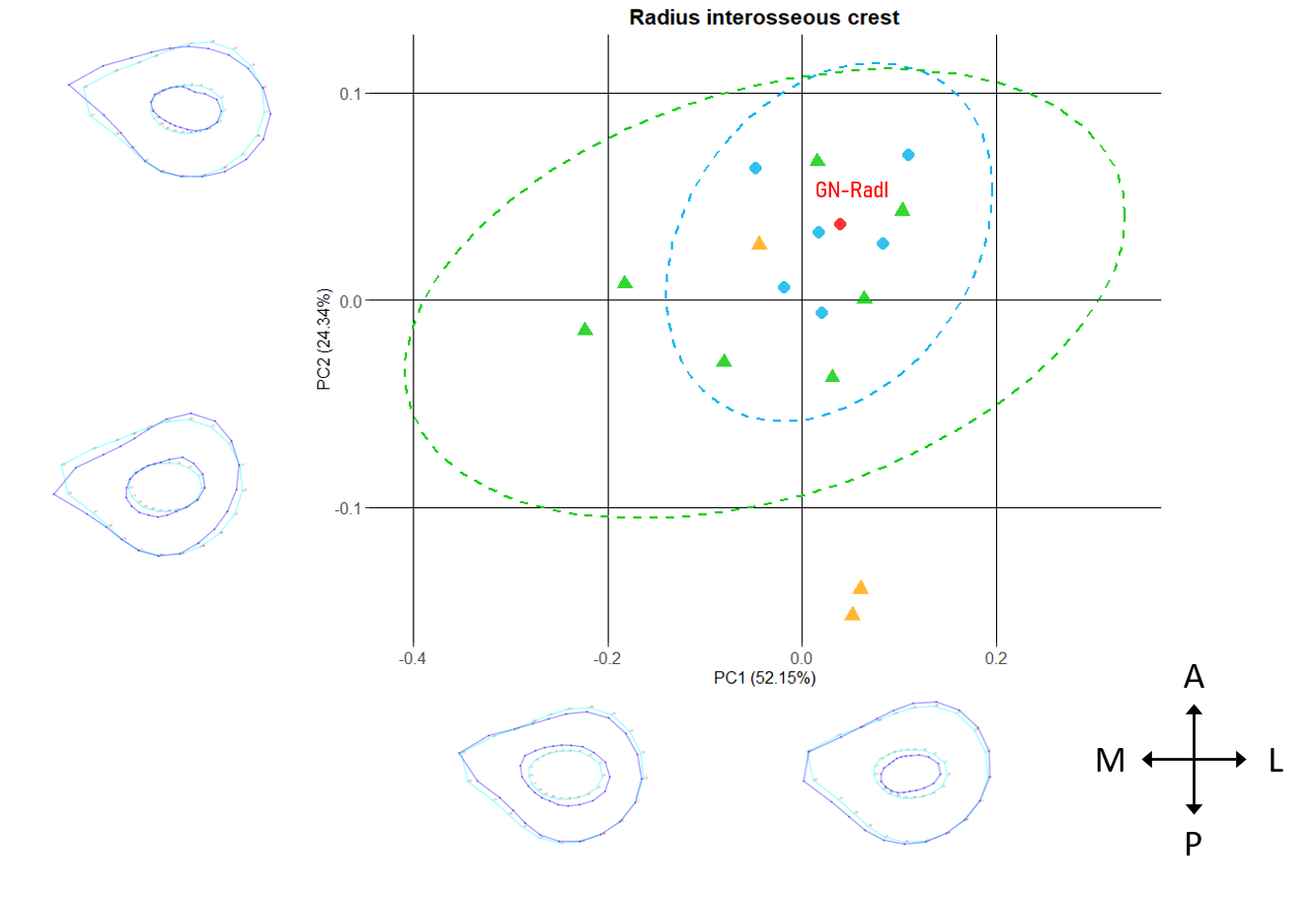


## **Supplementary Figure S3. Radial cross-sectional shape analysis based on Procrustes coordinates from the endosteal and periosteal contours at the maximum medial extension of the interosseous crest.**

The wireframes represent landmark configurations for PC scores of 0.1 (right on PC1 and top on PC2) and -0.1 (left on PC1 and bottom on PC2). Red diamonds: Goyet Neandertals; blue diamonds: Neandertals (N = 5); orange triangles: UPHS (N = 3); green triangles: Neolithic individuals (N = 7)*.* Ellipses represent the 95% confidence intervals of the comparative groups. A: anterior, L: lateral, P: posterior, M: medial.

# Supplementary Information References

1. Rohland, N., Glocke, I., Aximu-Petri, A. & Meyer, M. Extraction of highly degraded DNA from ancient bones, teeth and sediments for high-throughput sequencing. *Nature Protocols* **13**, 2447–2461 (2018).

2. Dabney, J. *et al.* Complete mitochondrial genome sequence of a Middle Pleistocene cave bear reconstructed from ultrashort DNA fragments. *Proceedings of the National Academy of Sciences* **110**, 15758–15763 (2013).

3. Gansauge, M.-T. & Meyer, M. Single-stranded DNA library preparation for the sequencing of ancient or damaged DNA. *Nature Protocols* **8**, 737–748 (2013).

4. Gansauge, M.-T., Aximu-Petri, A., Nagel, S. & Meyer, M. Manual and automated preparation of single-stranded DNA libraries for the sequencing of DNA from ancient biological remains and other sources of highly degradSed DNA. *Nature Protocols* **15**, 2279–2300 (2020).

5. Glocke, I. & Meyer, M. Extending the spectrum of DNA sequences retrieved from ancient bones and teeth. *Genome Research* **27**, 1230–1237 (2017).

6. Kircher, M., Sawyer, S. & Meyer, M. Double indexing overcomes inaccuracies in multiplex sequencing on the Illumina platform. *Nucleic Acids Research* **40**, e3 (2012).

7. Zavala, E. I. *et al.* Quantifying and reducing cross‐contamination in single‐ and multiplex hybridization capture of ancient DNA. *Molecular Ecology Resources* **22**, 2196–2207 (2022).

8. Church, D. M. *et al.* Modernizing Reference Genome Assemblies. *PLoS Biology* **9**, e1001091 (2011).

9. Li, H. & Durbin, R. Fast and accurate long-read alignment with Burrows–Wheeler transform. *Bioinformatics* **26**, 589-595 (2010).

10. Meyer, M. *et al.* A High-Coverage Genome Sequence from an Archaic Denisovan Individual. *Science* **338**, 222–226 (2012).

11. Peyrégne, S. & Peter, B. M. AuthentiCT: a model of ancient DNA damage to estimate the proportion of present-day DNA contamination. *Genome Biology* **21**, 246 (2020).

12. Rohland, N., Harney, E., Mallick, S., Nordenfelt, S. & Reich, D. Partial uracil–DNA–glycosylase treatment for screening of ancient DNA. *Philosophical Transactions of the Royal Society B: Biological Sciences* **370**, 20130624 (2015).

13. Fu, Q. *et al.* An early modern human from Romania with a recent Neanderthal ancestor. *Nature* **524**, 216–219 (2015).

14. Peltzer, A. *et al.* EAGER: efficient ancient genome reconstruction. *Genome Biology* **17**, 60 (2016).

15. Jónsson, H., Ginolhac, A., Schubert, M., Johnson, P. L. F. & Orlando, L. mapDamage2.0: fast approximate Bayesian estimates of ancient DNA damage parameters. *Bioinformatics* **29**, 1682–1684 (2013).

16. Skoglund, P. *et al.* Separating endogenous ancient DNA from modern day contamination in a Siberian Neandertal. *Proceedings of the National Academy of Sciences* **111**, 2229–2234 (2014).

17. Hajdinjak, M. *et al.* Reconstructing the genetic history of late Neanderthals. *Nature* **555**, 652–656 (2018).

18. Mittnik, A., Wang, C.-C., Svoboda, J. & Krause, J. A Molecular Approach to the Sexing of the Triple Burial at the Upper Paleolithic Site of Dolní Věstonice. *PLoS ONE* **11**, e0163019 (2016).

19. Wißing, C. *et al.* Isotopic evidence for dietary ecology of late Neandertals in North-Western Europe. *Quaternary International* **411**, 327–345 (2016).

20. Wißing, C. *et al.* Stable isotopes reveal patterns of diet and mobility in the last Neandertals and first modern humans in Europe. *Scientific Reports* **9**, 4433 (2019).

21. Rougier, H. *et al.* Neandertal cannibalism and Neandertal bones used as tools in Northern Europe. *Scientific Reports* **6**, 29005 (2016).

22. Bocherens, H., Fizet, M. & Mariotti, A. Diet, physiology and ecology of fossil mammals as inferred from stable carbon and nitrogen isotope biogeochemistry: implications for Pleistocene bears. *Palaeogeography, Palaeoclimatology, Palaeoecology* **107**, 213–225 (1994).

23. Fizet, M. *et al.* Effect of diet, physiology and climate on carbon and nitrogen stable isotopes of collagen in a late pleistocene anthropic palaeoecosystem: Marillac, Charente, France. *Journal of Archaeological Science* **22**, 67–79 (1995).

24. DeNiro, M. J. Postmortem preservation and alteration of in vivo bone collagen isotope ratios in relation to palaeodietary reconstruction. *Nature* **317**, 806–809 (1985).

25. Ambrose, S. H. Effects of diet, climate and physiology on nitrogen isotope abundances in terrestrial foodwebs. *Journal of Archaeological Science* **18**, 293–317 (1991).

26. Cowgill, L. W. The ontogeny of Holocene and Late Pleistocene human postcranial strength. *American Journal of Physical Anthropology* **141**, 16–37 (2010).

27. Cowgill, L. W., Warrener, A., Pontzer, H. & Ocobock, C. Waddling and toddling: The biomechanical effects of an immature gait. *American Journal of Physical Anthropology* **143**, 52–61 (2010).

28. Fotiadou, C. M. *et al.* Archaeogenetic insights into the demographic history of late Neanderthals (under review).

29. Bossoms Mesa, A. *et al.* Resolving the relatedness of the Neandertals from the Troisième caverne of Goyet using ancient DNA. *PaleoAnthropology* **2022**, 437 (2022).

30. Steele, D. G. & McKern, T. W. A method for assessment of maximum long bone length and living stature from fragmentary long bones. *American Journal of Physical Anthropology* **31**, 215–227 (1969).

31. Trinkaus, E. & Ruff, C. B. Femoral and Tibial Diaphyseal Cross-Sectional Geometry in Pleistocene Homo. *PaleoAnthropology* **2012**, 13–62 (2012).

32. Jacobs, K. Estimating femur and tibia length from fragmentary bones: An evaluation of Steele’s (1970) method using a prehistoric European sample. *American Journal of Physical Anthropology* **89**, 333–345 (1992).

33. Ruff, C. B. Long bone articular and diaphyseal structure in old world monkeys and apes. I: Locomotor effects. *American Journal of Physical Anthropology* **119**, 305–342 (2002).

34. Trinkaus, E. The Sexual Attribution of the La Quina 5 Neandertal. *Bulletins et mémoires de la Société d'Anthropologie de Paris* **28**, 111–117 (2016).

35. Brůžek, J., Santos, F., Dutailly, B., Murail, P. & Cunha, E. Validation and reliability of the sex estimation of the human os coxae using freely available DSP2 software for bioarchaeology and forensic anthropology. *American Journal of Physical Anthropology* **164**, 440–449 (2017).

36. Santos, F., Guyomarc’h, P., Rmoutilova, R. & Bruzek, J. A method of sexing the human os coxae based on logistic regressions and Bruzek’s nonmetric traits. *American Journal of Physical Anthropology* **169**, 435–447 (2019).

37. Rmoutilová, R. *et al.* Sex estimation of the adult Neandertal Regourdou 1 (Montignac, France): Implications for sexing human fossil remains. *Journal of Human Evolution* **189**, 103470 (2024).

38. Marchal, F. A new morphometric analysis of the hominid pelvic bone. *Journal of Human Evolution* **38**, 347–365 (2000).

39. Trinkaus, E. Sexual differences in Neanderthal limb bones. *Journal of Human Evolution* **9**, 377–397 (1980).

40. Ruff, C. Sexual dimorphism in human lower limb bone structure: relationship to subsistence strategy and sexual division of labor. *Journal of Human Evolution* **16**, 391–416 (1987).

41. Walker, M. J., Ortega, J., López, M. V., Parmová, K. & Trinkaus, E. Neandertal postcranial remains from the Sima de las Palomas del Cabezo Gordo, Murcia, southeastern Spain. *American Journal of Physical Anthropology* **144**, 505–515 (2011).

42. Heim, J.-L. *Les Hommes fossiles de La Ferrassie: squelette des membres*. (Masson, Paris New York Barcelone, 1982).

43. Fraipont, J. & Lohest, M. La race humaine de Néanderthal ou de Canstadt en Belgique. *Archives de Biologie* **7**, 587–755 (1887).

44. Hrdlička, A. The skeletal remains of early man. *Smithsonian Miscellaneous Collections* **83**, 1–379 (1930).

45. Genoves, S. The Problem of the Sex of Certain Fossil Hominids, with Special Reference to the Neandertal Skeletons from Spy. *The Journal of the Royal Anthropological Institute of Great Britain and Ireland* **84**, 131 (1954).

46. Hambücken, A. Étude du degré de robustesse des os longs du membre supérieur des Néandertaliens. *Bulletins et mémoires de la Société d'Anthropologie de Paris* **7**, 37–47 (1995).

47. Rougier, H. *et al.* Collections de la Grotte de Spy : (re)découvertes et inventaire anthropologique. *Notae Praehistoricae* **24**, 181–190 (2004).

48. Trinkaus, E. & Ruff, C. B. Diaphyseal cross-sectional morphology and biomechanics of the Fond-de-Forêt 1 femur and the Spy 2 femur and tibia. *Anthropologie et Préhistoire* **100**, 33–42 (1989).

49. Lalueza-Fox, C. *et al.* Genetic evidence for patrilocal mating behavior among Neandertal groups. *Proceedings of the National Academy of Sciences* **108**, 250–253 (2011).

50. Duveau, J., Berillon, G., Verna, C., Laisné, G. & Cliquet, D. The composition of a Neandertal social group revealed by the hominin footprints at Le Rozel (Normandy, France). *Proceedings of the National Academy of Sciences* **116**, 19409–19414 (2019).

51. Mafessoni, F. *et al.* A high-coverage Neandertal genome from Chagyrskaya Cave. *Proceedings of the National Academy of Sciences* **117**, 15132–15136 (2020).

52. Mayoral, E. *et al.* Tracking late Pleistocene Neandertals on the Iberian coast. *Scientific Reports* **11**, 4103 (2021).

53. Skov, L. *et al.* Genetic insights into the social organization of Neanderthals. *Nature* **610**, 519–525 (2022).

54. Ledermann, S. *Nouvelles tables-types de mortalité*. (Presses universitaires de France, 1969).

55. Bocquentin, F. Pratiques funéraires, paramètres biologiques et identités culturelles au Natoufien: une analyse archéo-anthropologique. (Université Bordeaux I, Talence, 2003).

56. Masset, C. Le ‘recrutement’ d’un ensemble funéraire. in *Anthropologie physique et archéologie. Méthodes d’étude des sépultures* 111–134 (Paris, 1987).

57. Sellier, P. Paléodémographie et archéologie funéraire : les cimetières de Mehrgarh, Pakistan. *PALEO* **21**, 123–143 (1995).

58. Rosas, A. *et al.* Identification of Neandertal individuals in fragmentary fossil assemblages by means of tooth associations: The case of El Sidrón (Asturias, Spain). *Comptes Rendus Palevol* **12**, 279–291 (2013).

59. Reimer, P. J. *et al.* The IntCal20 Northern Hemisphere Radiocarbon Age Calibration Curve (0–55 cal kBP). *Radiocarbon* **62**, 725–757 (2020).

60. Bronk Ramsey, C. Bayesian Analysis of Radiocarbon Dates. *Radiocarbon* **51**, 337–360 (2009).
